# Effect of amino acid mutations on the conformational dynamics of amyloidogenic immunoglobulin light-chains: A combined NMR and *in silico* study

**DOI:** 10.1038/s41598-017-10906-w

**Published:** 2017-09-04

**Authors:** Sujoy Mukherjee, Simon P. Pondaven, Kieran Hand, Jillian Madine, Christopher P. Jaroniec

**Affiliations:** 1 Department of Chemistry and Biochemistry, The Ohio State University, Columbus, Ohio, 43210 USA; 2 Structural Biology and Bio-Informatics Division, Indian Institute of Chemical Biology, Kolkata, 700032 India; 3 Institute of Integrative Biology, University of Liverpool, Liverpool, UK

## Abstract

The conformational dynamics of a pathogenic κ4 human immunoglobulin light-chain variable domain, SMA, associated with AL amyloidosis, were investigated by ^15^N relaxation dispersion NMR spectroscopy. Compared to a homologous light-chain, LEN, which differs from SMA at eight positions but is non-amyloidogenic *in vivo*, we find that multiple residues in SMA clustered around the N-terminus and CDR loops experience considerable conformational exchange broadening caused by millisecond timescale protein motions, consistent with a destabilized dimer interface. To evaluate the contribution of each amino acid substitution to shaping the dynamic conformational landscape of SMA, NMR studies were performed for each SMA-like point mutant of LEN followed by *in silico* analysis for a subset of these proteins. These studies show that a combination of only three mutations located within or directly adjacent to CDR3 loop at the dimer interface, which remarkably include both destabilizing (Q89H and Y96Q) and stabilizing (T94H) mutations, largely accounts for the differences in conformational flexibility between LEN and SMA. Collectively, our studies indicate that a correct combination of stabilizing and destabilizing mutations is key for immunoglobulin light-chains populating unfolded intermediates that result in amyloid formation, and underscore the complex nature of correlations between light-chain conformational flexibility, thermodynamic stability and amyloidogenicity.

## Introduction

Plasma cell dyscrasias are a diverse group of disorders associated with proliferation of clonal plasma cells, and, in certain cases, secretion of large quantities of immunoglobulin light-chain variable domain proteins (Ig V_L_s) into the bloodstream. Most Ig V_L_s are excreted in urine as Bence-Jones proteins, but a fraction can misfold and aggregate into fibrillar amyloid plaques. Deposition of these amyloid plaques in the extracellular space of critical organs and tissues is responsible for amyloid light-chain (AL) amyloidosis, one of the most common forms of systemic amyloidosis in humans^1–3^. The kidneys and heart are among the primary organs affected by AL amyloidosis, and in case of the latter the disease manifests itself as rapidly progressive congestive heart failure with median life expectancy of less than a year following diagnosis^3–5^.

SMA is a 114 amino acid (aa) residue Ig V_L_ protein of the κ4 sub-type, isolated from lymph node-derived amyloid plaques of a patient suffering from AL amyloidosis^6^. The protein contains a total of eight aa mutations (S29N, K30R, P40L, Q89H, T94H, Y96Q, S97T and I106L) relative to a homologous 114-residue κ4 Ig V_L_, LEN, that differs from the germline κ4 sequence at a single position (N29S) but is non-amyloidogenic *in vivo*, having been extracted as a Bence-Jones protein from a multiple myeloma patient with no symptoms of renal dysfunction or AL amyloidosis^7^. Multiple studies have revealed that mutations to germline Ig V_L_ sequences could produce thermodynamically unstable light-chains with higher propensity to aggregate through partially unfolded intermediates and that non-amyloidogenic Ig V_L_s could be converted to fibrils *in vitro* upon suitable destabilization, and have attempted to elucidate the roles of individual mutations in conferring amyloidogenicity to Ig V_L_ domains^8–15^. For the LEN and SMA light-chains, Raffen *et al*.^10^ have used a GdnHCl unfolding assay to determine the difference in their thermodynamic stabilities (ΔΔ*G*_*unf*_), as well as the ΔΔ*G*_*unf*_ values for each of the eight SMA-like mutants of LEN. They found that the P40L, Q89H and Y96Q mutations destabilized LEN by 0.7, 1.0 and 3.2 kcal/mol, respectively, while the S29N, T94H and S97T substitutions were stabilizing with ΔΔ*G*_*unf*_ values ranging from −1.0 to −0.6 kcal/mol and the K30R (ΔΔ*G*_*unf*_ = 0.1 kcal/mol) and I106L (ΔΔ*G*_*unf*_ = −0.2 kcal/mol) mutants had stabilities comparable to native LEN. Furthermore, the effects of the SMA-like mutations were found to be non-cooperative, given that the combined ΔΔ*G*_*unf*_ of 2.5 kcal/mol for the eight individual point mutants of LEN was virtually identical to the 2.6 kcal/mol value determined for SMA^10^. While it is generally accepted that the overall destabilization of Ig V_L_s (and indeed numerous other proteins)^16^ correlates broadly with increased aggregation propensity^8–15^, the atomic-level mechanistic details of how mutations of specific amino acid residues promote amyloid formation often remain unclear and the correspondence between protein thermodynamic stability and ability to form amyloid is certainly not one-to-one. For instance, the SMA-like P40L mutant of LEN, which is considerably more thermodynamically stable than the Q89H and Y96Q mutants, was found to form fibrils *in vitro* more readily than both LEN Q89H and LEN Y96Q^10, 17^. In addition, another mutant of LEN, which was designed to mimic an analogous patient-derived κ4 Ig V_L_ (REC)^10^, was reported to be non-amyloidogenic in spite of having nearly 4 kcal/mol lower stability than LEN (and 1.1 kcal/mol lower than the highly amyloidogenic SMA).

Recombinant SMA and LEN light-chains have been extensively investigated by using biochemical and biophysical methods by the groups of Stevens^7, 10, 18–21^ and Fink^22–28^, and the high-resolution X-ray crystal structure of LEN (PDB entry 1LVE) is also known^29^. These experiments have revealed that under physiological conditions both SMA and LEN are present in dimeric form^7^, with SMA being strongly amyloidogenic and LEN stable in solution^7, 10, 22^. Further systematic *in vitro* protein aggregation studies have shown that in the presence of denaturants^10, 23^ or at pH 2 with vigorous agitation^24–26^ LEN could also be converted to amyloid, concluding altogether that destabilization of the protein dimer interface is the most likely first step en route to assembly of LEN and SMA Ig V_L_s into fibrils. However, while the aforementioned studies have provided important insights about the global conformational landscapes and misfolding properties of SMA and LEN light-chains, they employed intrinsically low-resolution techniques and thus were unable to probe the links between the structural and dynamic features, thermodynamic stability and aggregation behaviour of these proteins at the level of individual residues.

Building upon these earlier studies, we have recently applied multidimensional solution-state nuclear magnetic resonance (NMR) techniques to the LEN Ig V_L_^30, 31^ aimed at quantitatively assessing the extent of protein structural and dynamic changes as a function of pH with site-specific resolution; note that analogous NMR-based approaches have also been used to investigate other amyloid-forming Ig V_L_s^12, 32–34^. Specifically, we were able to show that, on the whole, LEN retains its native dimeric three-dimensional fold under physiological (pH 6.5) and acidic (pH 2) conditions. At the same time, however, by using relaxation dispersion NMR methods^35, 36^ which permit characterization of low-populated protein conformational states existing in dynamic equilibrium with a predominant ground state, we found that a subset of ~30 aa residues, most co-localized at or near the dimer interface, exhibit significant millisecond time scale backbone motions at both pH values that are consistent with the presence of excited state conformers for LEN. Most remarkably, these experiments revealed a ca. 3–4-fold increase in the population of these excited state protein conformers between physiological and acidic pH (from ~2–4% to ~10–15%), with concomitant increase in dynamics for most residues involved in interactions that stabilize the dimer interface^31^. In the present study we extend this NMR methodology toward the amyloidogenic SMA protein in order to understand how its structure and dynamics contrast with those of LEN and to identify which of the eight amino acid substitutions that distinguish these two homologous proteins are responsible for any differences in the conformational dynamics between them. To provide further atomistic insights into the correlations between conformational flexibility, thermodynamic stability and amyloidogenicity of these light-chain variable domains the NMR experiments were complemented by *in silico* analysis of several point mutants of LEN.

## Results

### Effect of Amino Acid Mutations on Ig V_L_ Backbone Structure

Two dimensional ^15^N-^1^H heteronuclear single quantum coherence (HSQC) NMR spectra were recorded for SMA and each of the eight SMA-like point mutants of LEN at pH 6.5. Five of the mutants, including S29N, K30R, P40L, S97T and I106L, yielded spectra that were virtually superimposable with the spectrum of LEN (see Supplementary Fig. S1), with only minor differences in the ^15^N and/or ^1^H resonance frequencies observed for several residues directly adjacent or in close spatial proximity to the mutation sites. These results unequivocally indicate that the S29N, K30R, P40L, S97T and I106L mutants adopt the native LEN fold, and for these proteins over 95% of the ^15^N-^1^H correlations could be unambiguously identified by directly mapping the chemical shift assignments previously established for LEN^30^.

**Figure 1:**
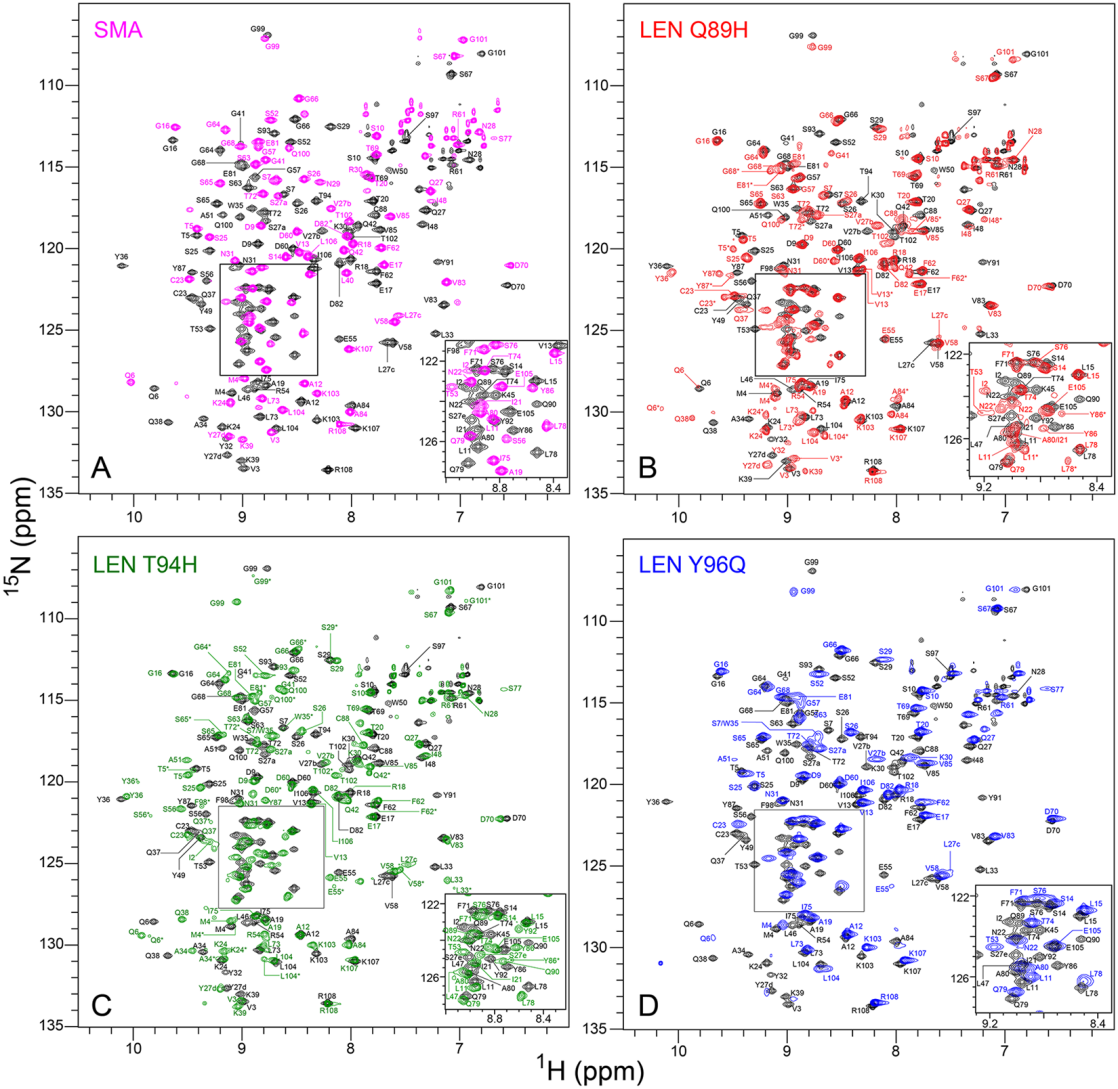
Two dimensional ^15^N-^1^H HSQC spectra of ^15^N-labeled (**A**) SMA (magenta), (**B**) LEN Q89H (red), (**C**) LEN T94H (green), and (**D**) LEN Y96Q (blue) recorded at 800 MHz ^1^H frequency and 25 °C, overlaid on the spectrum of LEN (black). The spectra for all other SMA-like mutants of LEN mutants were virtually identical to LEN (see Supplementary Fig. S1). The backbone amide resonance assignments are indicated in corresponding colors.

**Figure 2:**
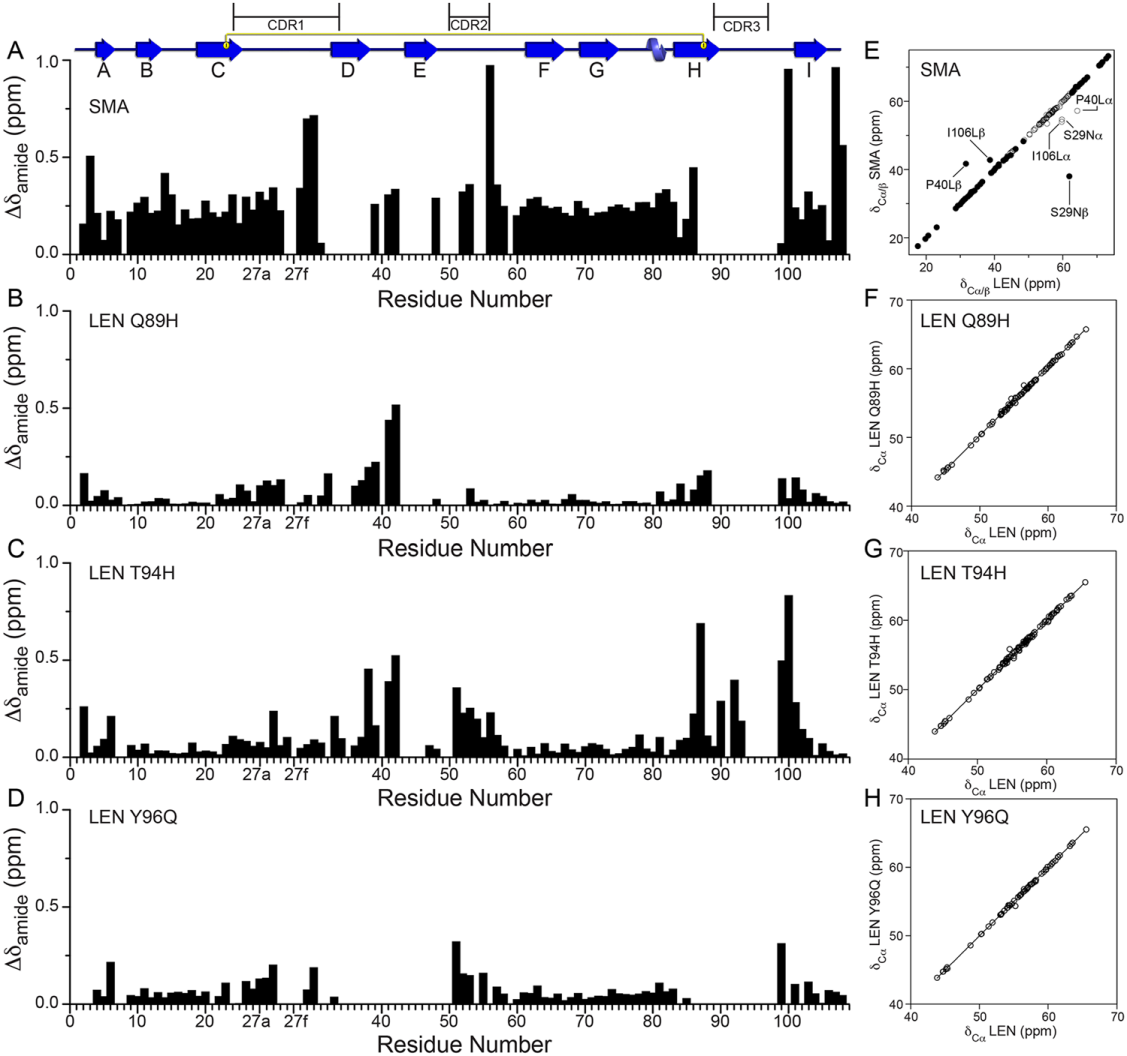
(**A**–**D**) Plots of residue-specific amide chemical shift changes between LEN and (**A**) SMA, (**B**) LEN Q89H, (**C**) LEN T94H, and (**D**) LEN Y96Q. (**E**–**H**) Plots of ^13^Cα chemical shifts for LEN versus (**E**) SMA, (**F**) LEN Q89H, (**G**) LEN T94H, and (**H**) LEN Y96Q (open circles). For SMA the ^13^Cβ chemical shifts are also compared to LEN (filled circles).

In contrast, the ^15^N-^1^H spectra of SMA and the Q89H, T94H and Y96Q mutants of LEN targeting complementarity determining region 3 (CDR3) were found to differ more significantly from the spectrum of LEN at pH 6.5 as shown in Fig. 1, and also from that recorded at pH 2 in our earlier study^31^. Sequential resonance assignments, established using ^13^C, ^15^N-labeled proteins as described in the Methods section, enabled unambiguous assignments of 80 of 109 non-proline residues for SMA and 84, 97 and 68 of 108 non-proline residues for LEN Q89H, T94H and Y96Q, respectively (see Supplementary Tables S1–S2). The vast majority of the residues that could not be assigned were clustered in and around the CDR1, CDR2 and CDR3 loops and broadened beyond detection due to conformational exchange dynamics similar to those observed for LEN under acidic conditions^31^. Additionally, for LEN Q89H and T94H discernible resonance doubling was observed for a few residues (e.g., Q6 and L104; indicated by asterisks), indicating the existence of two distinct protein conformers in slow exchange at these sites. For each residue that could be assigned for SMA and LEN Q89H, T94H and Y96Q, the amide chemical shift perturbations with respect to LEN were calculated according to:

$${\rm{\Delta }}{\delta }_{amide}=\,\sqrt{{({\rm{\Delta }}{\delta }_{H})}^{2}+{({\rm{\Delta }}{\delta }_{N}/5)}^{2}\,},$$

where ${\rm{\Delta }}{\delta }_{H}$ and ${\rm{\Delta }}{\delta }_{N}$ are the differences in amide ^1^H and ^15^N chemical shifts between LEN and the protein of interest. The plots of ${\rm{\Delta }}{\delta }_{amide}$ as a function of residue number are shownin Fig. 2A–D, and indicate, not surprisingly, that SMA with its eight mutations relative to LEN displays the largest amide chemical shift perturbations that are distributed throughout the entire protein. Quantitative analysis of these data revealed average ${\rm{\Delta }}{\delta }_{amide}$ values of 0.29 ± 0.18 ppm for SMA, 0.06 ± 0.08 ppm for LEN Q89H, 0.12 ± 0ppm for LEN T94H, and 0.09 ± 0.19 ppm for LEN Y96Q (for comparison, the average ${\rm{\Delta }}{\delta }_{amide}$ for LEN at pH 2 vs. 6.5 was found to be 0.17 ± 0.17 ppm in our earlier study)^31^.

Given that amide ^15^N and ^1^H chemical shifts are in general highly sensitive to multiple factors, the perturbations in these shifts relative to LEN observed for SMA and the three SMA-like mutants of LEN are not necessarily indicative of widespread protein structural changes. Indeed, in our previous study of LEN^31^ we found that in spite of considerable amide chemical shift perturbations between pH 6.5 and 2 the changes in the corresponding backbone ^13^C chemical shifts, which depend strongly on the conformation of the protein backbone^37^, were in fact minimal. A similar analysis was performed here for SMA and LEN Q89H, T94H and Y96Q and is presented in Fig. 2E–H. For all four proteins, this analysis reveals negligible changes in the ^13^Cα (and for SMA also ^13^Cβ) shifts relative to LEN for the detectable residues that are not strongly influenced by conformational exchange phenomena (all ^13^C shift correlations had R^2^ values >0.99 with the mutated residues excluded). Altogether, the comparisons of ^1^H, ^15^N and/or ^13^C NMR chemical shifts for LEN, SMA and all SMA-like LEN mutants indicate that at physiological pH all these κ4 Ig V_L_s have very similar backbone folds for the β-sheet rich framework regions. Concurrently, more localized structural and dynamic changes are possible, in particular for SMA and the Q89H, T94H and Y96Q LEN mutants, for residues located in the CDR1, CDR2 and CDR3 loops undergoing the most significant conformational exchange. These results also suggest that differences in the details of hydrogen bonding coupled to protein conformational dynamics, which are discussed below, are most likely responsible for the observed chemical shift perturbations in the ^15^N-^1^H HSQC spectra of SMA and LEN Q89H, T94H and Y96Q relative to LEN.

### Conformational Dynamics of SMA and SMA-Like Mutants of LEN

Prior to investigating the slow conformational exchange dynamics of SMA and SMA-like mutants of LEN, which are of primary interest in this study given our earlier findings for LEN^31^, we sought to confirm the oligomeric state of these proteins under the conditions used for our NMR experiments. First, we have employed the well-established near-UV circular dichroism based assay^38^, which can yield the monomer-dimer equilibrium constants for Ig V_L_s from a non-linear fit of the observed molar ellipticity at 282.5 nm as a function of protein concentration and previously has been successfully applied to LEN^24^, to rapidly screen all the proteins of interest. These measurements yielded association constants ranging from ~10^5^ to ~10^7^ M^−1^ for the different light-chains, with majority falling between 10^5^ and 10^6^ M^−1^ (see Supplementary Fig. S2 and Table S3), in good agreement with previous studies^7, 24^. Furthermore, for SMA we have determined the residue-specific longitudinal relaxation (*R*_1_), transverse relaxation (*R*_2_) and steady-state heteronuclear {^1^H}-^15^N nuclear Overhauser enhancement (NOE) parameters for the backbone ^15^N nuclei (Supplementary Fig. S3). The *R*_1_ and NOE values for SMA mirrored those previously determined for LEN, while the *R*_2_ values for SMA exceeded the LEN values by ~20% on average. Analysis of the ^15^N *R*_2_/*R*_1_ ratios^39^ yielded an estimated rotational correlation time of 15.0 ± 0.1 ns for SMA, which is similar to the ~13.5 ns correlation time obtained previously for LEN^31^ and consistent with the presence of a dimeric species for this ~12.8 kDa protein. In summary, at the ~1–2 mM concentrations used for the NMR measurements, SMA and all SMA-like mutants of LEN are present in solution predominantly as dimers.

**Figure 3:**
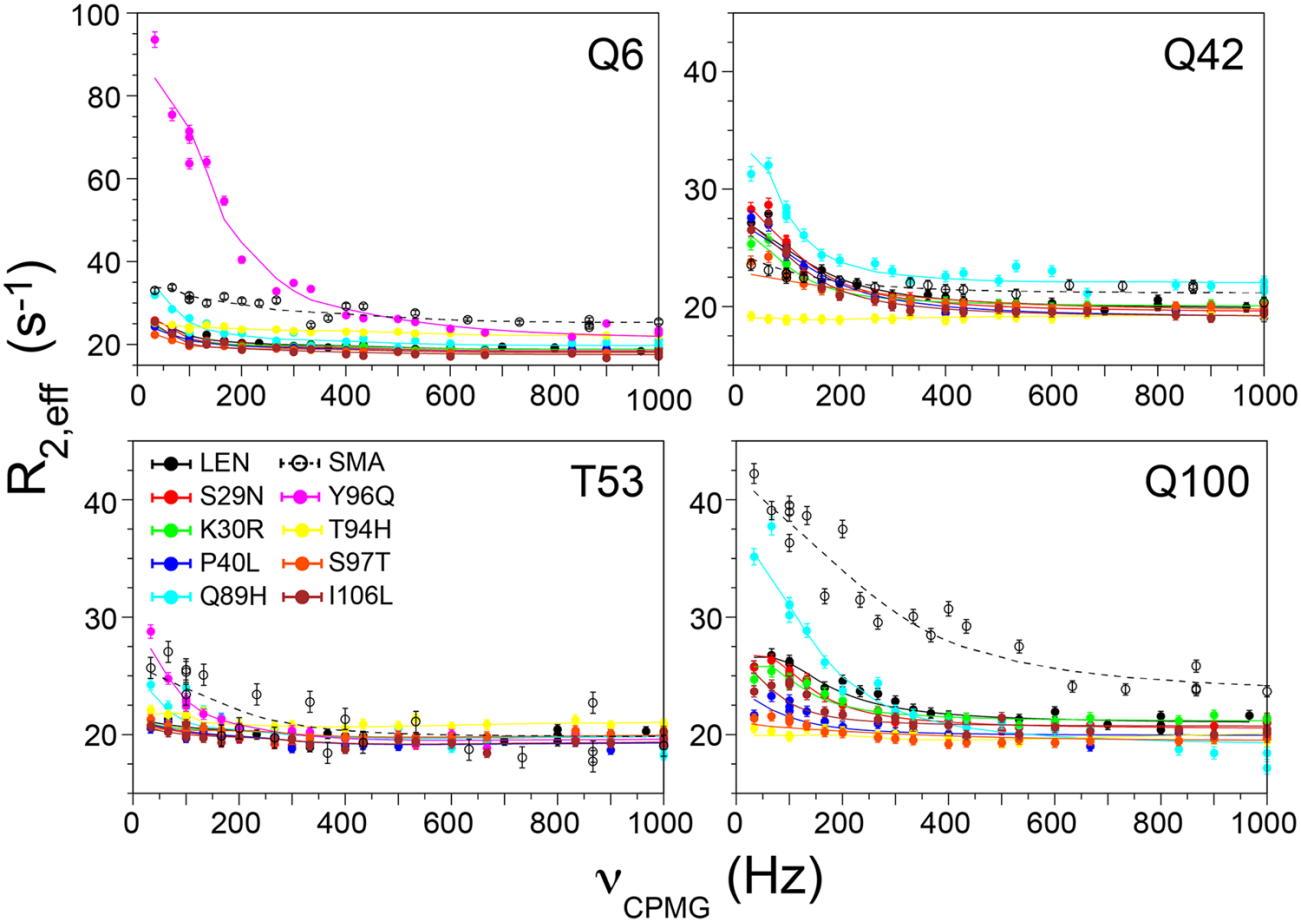
^15^N CPMG relaxation dispersion trajectories at 600 MHz ^1^H frequency for representative residues located in three regions of LEN, SMA and the eight SMA-like mutants of LEN displaying elevated conformational dynamics on the millisecond timescale (circles; see inset for details). Shown in lines of the corresponding color are calculated trajectories obtained by the global fitting of all residues from each dynamic region as described in the text.

**Figure 4:**
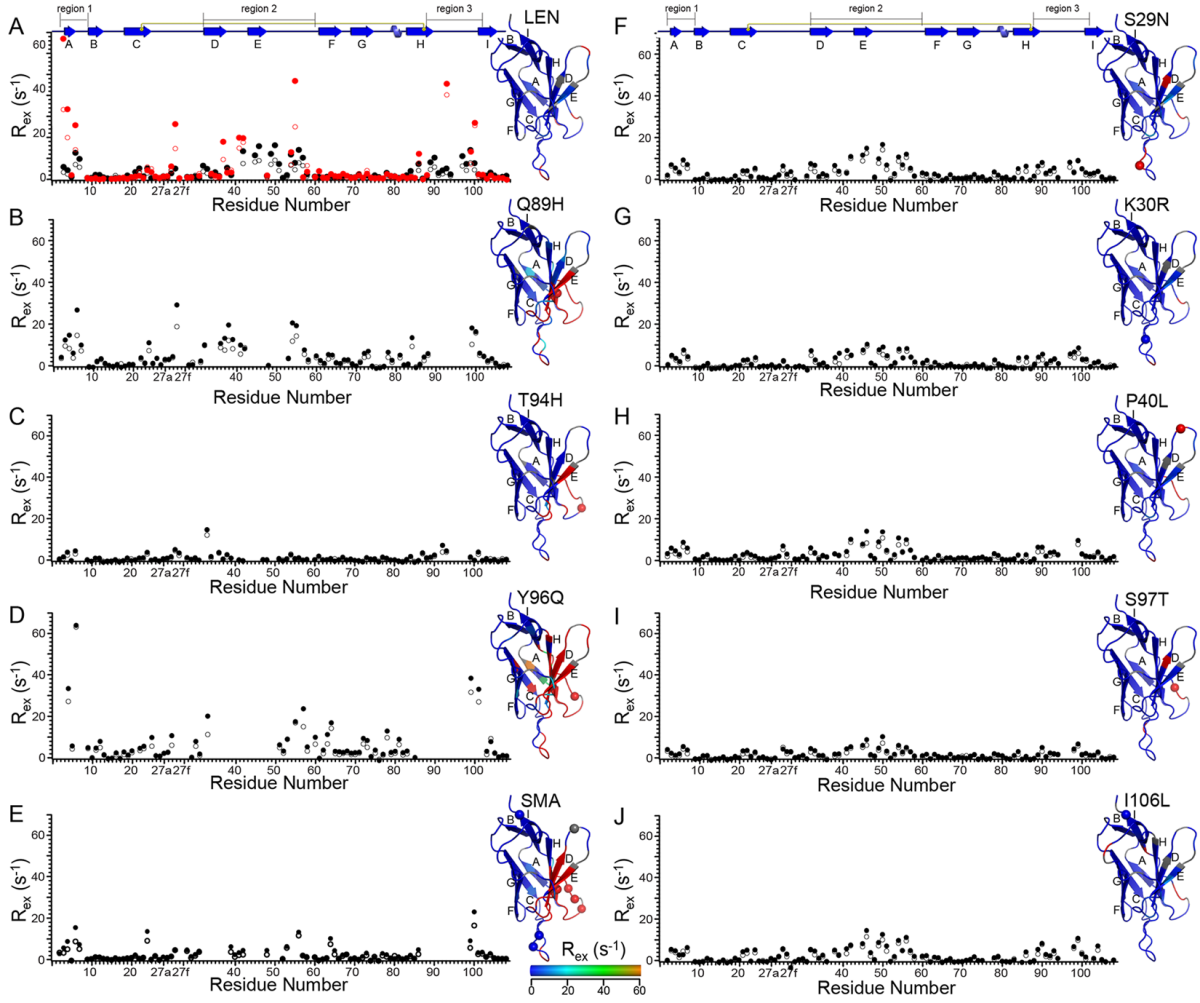
Plots of chemical exchange contribution to ^15^N transverse relaxation, *R*_*ex*_, at 600 MHz (open circles) and 800 MHz (filled circles) ^1^H frequency as a function of residue number for (**A**) LEN, (**B**) LEN Q89H, (**C**) LEN T94H, (**D**) LEN Y96Q, (**E**) SMA, (**F**) LEN S29N, (**G**) LEN K30R, (**H**) LEN P40L, (**I**) LEN S97T, and (**J**) LEN I106L. Previously reported data for LEN at pH 2^31^ are also shown in panel (**A**) for reference (open and filled red circles). For each residue, the relaxation trajectories at 600 and 800 MHz ^1^H frequency were fit simultaneously using the *CATIA* program as described in the text and the *R*_*ex*_ values were calculated as differences in the ^15^N *R*_*2*_ values calculated for the lowest and highest CPMG frequencies. For each protein, the *R*_*ex*_ values at 600 MHz ^1^H frequency are mapped onto the X-ray structure for one of the monomer subunits of LEN (PDB entry 1LVE), with magnitudes indicated by the color bar. Residues that were not detectable in the ^15^N-^1^H correlation spectra due to excessive conformational exchange broadening are indicated in red, and those excluded from the calculations due to signal overlap (and prolines) are indicated in grey. For all proteins, the amino acid differences relative to LEN are shown as spheres for the Cα atoms.

**Figure 5:**
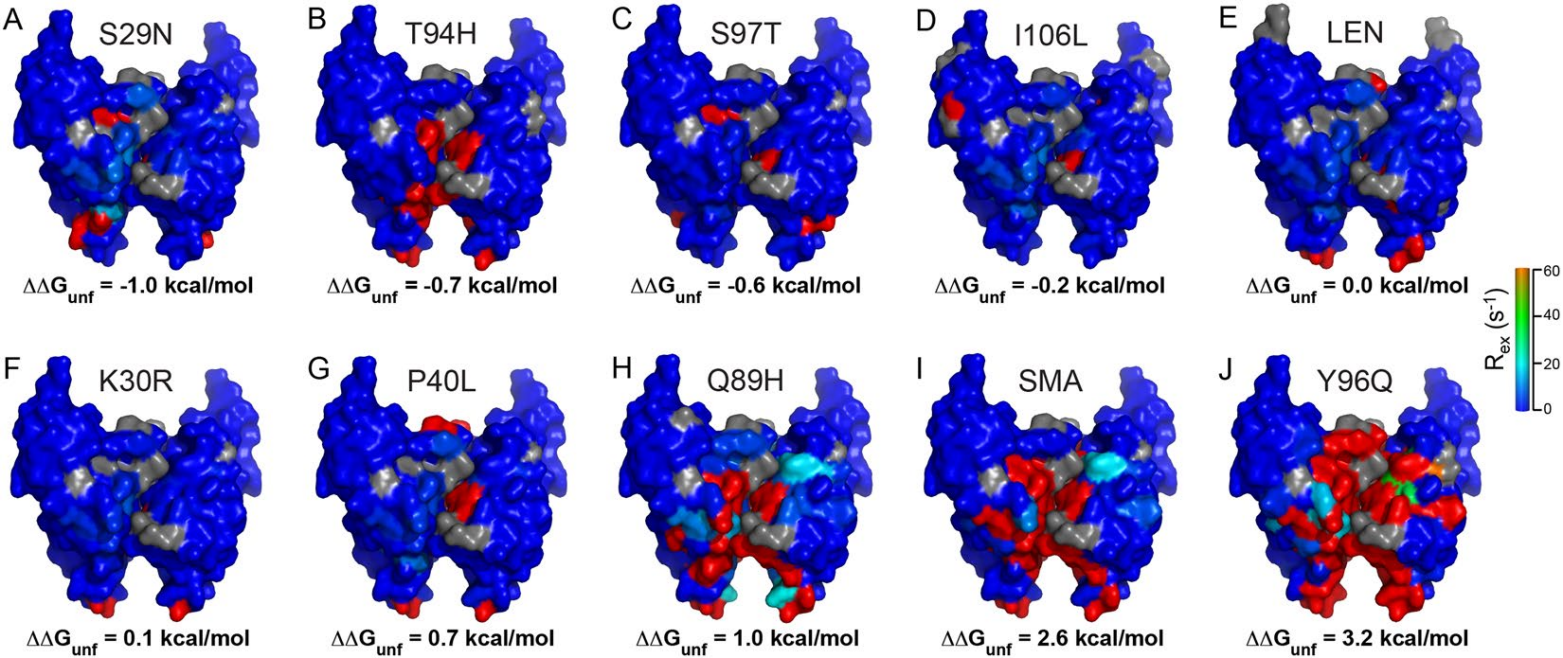
Surface representations of LEN, SMA and the eight SMA-like mutants of LEN in dimer form, depicting the locations of the most dynamic residues. The proteins are arranged in the order of decreasing thermodynamic stability with respect to LEN as reported by Raffen *et* *al*.^10^, with LEN S29N (**A**) being the most stable and Y96Q (**J**) the most unstable. The *R*_*ex*_ values obtained at 600 MHz ^1^H frequency are mapped onto the X-ray structure of LEN with color coding similar to that used in Fig. 4.

To assess the extent of slow, millisecond time scale protein backbone motions for the different Ig V_L_s we carried out ^15^N Carr-Purcell-Meiboom-Gill (CPMG) relaxation dispersion measurements at 14.1 and 18.8 T, corresponding to ^1^H frequencies of 600 and 800 MHz, respectively. Representative residue-specific trajectories of the effective ^15^N transverse relaxation rate, *R*_2,*eff*_, versus CPMG frequency, *ν*_*CPMG*_, recorded at 14.1 T for LEN, SMA and the eight SMA-like mutants of LEN are shown in Fig. 3. Additional ^15^N CPMG relaxation dispersion data were collected as controls at lower protein concentrations for the most and least thermodynamically stable mutants of LEN (S29N and Y96Q) to confirm that these data report exclusively on protein backbone dynamics and are not influenced by monomer-dimer exchange phenomena (Supplementary Figs S4 and S5). Initially, the trajectories were independently fit for individual residues to a two-site exchange model^40^, and the estimated chemical exchange contributions to transverse relaxation, *R*_*ex*_, were obtained as differences between simulated *R*_2,*eff*_ values for the lowest and highest CPMG frequencies. Figure 4 shows these *R*_*ex*_ values as a function of residue number and mapped onto the three-dimensional structure of LEN monomer (Fig. 4) and dimer (Fig. 5) for SMA and each SMA-like mutant of LEN (additionally, Supplementary Fig. S6 shows correlation plots of *R*_*ex*_ values for all the Ig V_L_s relative to LEN); the *R*_*ex*_ values reported in our earlier study for LEN at pH 6.5 and 2^31^ are also included for comparison in Fig. 4A (red circles). A broad overview of these data indicates that, in analogy to LEN^31^, most of the other κ4 Ig V_L_s investigated here display significant exchange contributions to ^15^N transverse relaxation (classified as *R*_*ex*_ > 3 s^−1^ at 600 MHz or the corresponding ^15^N-^1^H correlation being broadened beyond detection in the HSQC spectrum) that are primarily clustered in three distinct regions (denoted as regions 1, 2 and 3). Region 1 contains ~9 N-terminal amino acids, region 2, which has variable length for the different light-chains, is located between residues ~35–60 and encompasses FR2 framework region β-strand E and CDR2, and region 3 spans residues ~88–102 and includes CDR3 (c.f., Supplementary Fig. S8).

The residue specific *R*_*ex*_ profiles for the S29N, K30R, P40L, S97T and I106L mutants of LEN (Fig. 4F–J), which have ^15^N-^1^H HSQC NMR spectra that mirror that of LEN, on the whole resemble the profile for LEN at pH 6.5, with the K30R and S97T mutants showing slightly subdued conformational exchange dynamics relative to LEN and the *R*_*ex*_ profiles for S29N, P40L and I106L mutants being effectively identical to LEN (c.f., Supplementary Fig. S6). Particularly noteworthy is the finding that the substitution of leucine for proline at position 40—which was expected to lead to increased backbone mobility given that residue P40 is considered to be a stabilizer of the FR2 region^10, 17^—appears to have negligible impact on the protein dynamics. On the other hand, SMA and the Q89H, T94H and Y96Q mutants of LEN show *R*_*ex*_ profiles (Fig. 4B–E) that are clearly distinct from LEN and also from one another to varying degrees. Specifically, relative to LEN at pH 6.5, SMA, LEN Q89H and LEN Y96Q all display major conformational exchange broadening leading to complete suppression of NMR signals and/or elevated *R*_*ex*_ values for residues in regions 1–3 as well as for additional residues located outside of these regions in the case of the Q89H and Y96Q LEN mutants, with the largest effects observed for LEN Y96Q—moreover, the *R*_*ex*_ profiles for these three proteins are qualitatively broadly reminiscent of the profile observed for LEN at pH 2 (Fig. 4A, red circles). Interestingly, in contrast to all the other Ig V_L_s investigated in this study, for the T94H mutant of LEN the majority of millisecond timescale motions associated with conformational exchange broadening appear to have been effectively quenched with the possible exception of several residues located in region 3 and a few additional sites distributed throughout the protein (e.g., Y27d and L33).

More quantitative dynamic parameters, including population of the minor conformer (*p*_*B*_) and exchange rate (*k*_*ex*_), for each of the three regions undergoing the most significant conformational exchange were extracted, where possible, for the different light-chains by simultaneously fitting the ^15^N CPMG relaxation dispersion trajectories at 14.1 and 18.8 T for the relevant residues to the two-site exchange model. Modelling of the dynamics for a contiguous stretch of the protein sequence with a single exchange rate and minor conformer fraction is justified given that any partial unfolding of a part of a protein is likely to happen simultaneously for multiple adjacent residues rather than individual residues experiencing completely uncorrelated motions. Representative calculated trajectories corresponding to this fitting procedure are shown in Fig. 3, and the fitting results are summarized in Supplementary Table S4. These data indicate that for the S29N, K30R, P40L, S97T and I106L mutants of LEN the minor conformer populations in regions 1–3 are generally below ~4% and on par with wild-type LEN^31^, with the exception of region 1 for several of the mutants where somewhat higher (~8–10%) minor conformer populations are estimated. On the other hand, SMA and the most thermodynamically unstable LEN mutants (Q89H and Y96Q) are, for the most part, associated with minor conformer populations that are at least ~2–3-fold larger than native LEN and its stable mutants or exhibit such major conformational exchange broadening of the associated resonances that the dynamic parameters cannot be quantitatively estimated at all. Altogether the ^15^N CPMG relaxation dispersion measurements suggest that the conformational exchange dynamics in SMA and SMA-like mutants of LEN occur primarily along the dimer interface, and that the extent of these protein backbone motions, as reflected in the broadening and attenuation of NMR signals and/or increased populations of minor conformers, is reasonably strongly correlated with the decreasing thermodynamic stability of the different Ig V_L_ domains (Fig. 5 and Supplementary Fig. S7). Finally, we reiterate here that the ^15^N CPMG relaxation dispersion data in this study report on millisecond timescale protein conformational dynamics within the Ig V_L_ dimers, as opposed to monomer-dimer exchange phenomena which likely occur on slower timescales. This is established by the fact that measurements on concentrated and two-fold diluted protein samples, the latter of which are associated with significantly (~40–50%) higher monomer concentrations, yield virtually identical residue-specific trajectories of the effective ^15^N transverse relaxation rate versus CPMG frequency, in particular for residues showing large relaxation dispersions (Supplementary Figs S4 and S5). Furthermore, no correlations exist between the experimental *R*_*ex*_ profiles (Fig. 4), which differ widely from minimal conformational exchange (LEN T94H) to very significant exchange (LEN Y96Q), and the estimated monomer-dimer association constants, most of which are in the ~10^5^–10^6^ M^−1^ regime and similar for the κ4 Ig V_L_s in this study (Supplementary Fig. S2 and Table S3).

### *In Silico* Analysis of SMA-Like Mutants of LEN

To further investigate the impact of the K30R, P40L, T94H, Q89H and Y96Q substitutions on the structure of LEN, mutants were generated *in silico* using the LEN crystal structure as a template with Rosetta based energy-minimization. The lowest energy structure for each mutation was taken for further analysis. The LEN mutants selected for the *in silico* analysis included those for which the fingerprint ^15^N-^1^H HSQC NMR spectra and conformational exchange dynamics (c.f., Figs 1, 4 and 5) differed most significantly relative to LEN (i.e., T94H, Q89H and Y96Q) as well as P40L, which appears to be somewhat of an outlier as noted above in the sense that it displays decreased thermodynamic stability and increased propensity to form amyloid relative to LEN^10, 17^ but little, if any, structural or dynamic perturbations according to the NMR analysis. The K30R mutant with thermodynamic stability, structure and dynamics that are nearly identical to LEN was included as a control for comparative purposes.

**Figure 6:**
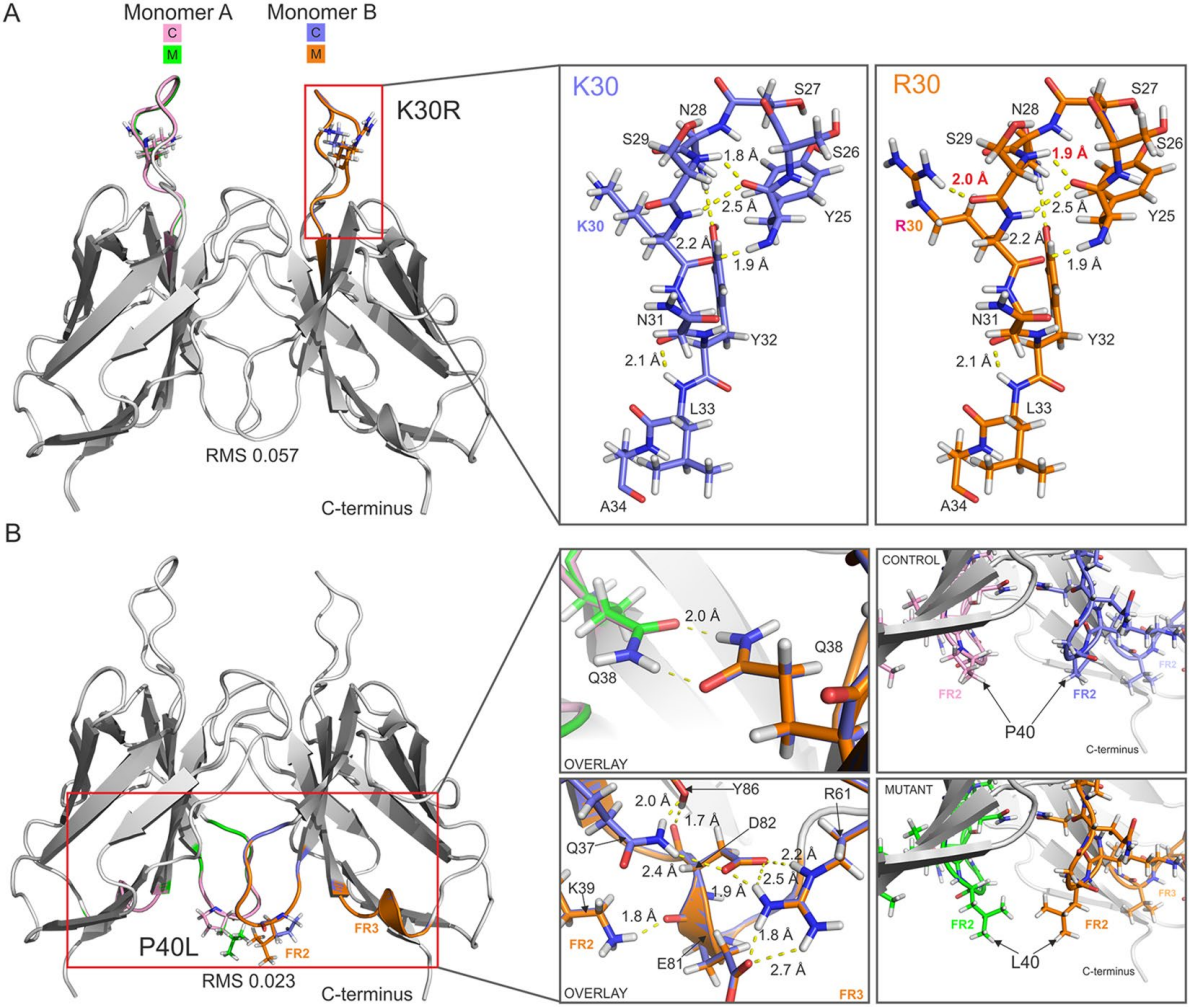
Computational analysis of mutation-induced structural changes for LEN K30R and P40L. Dimer structure of native LEN is shown as a cartoon overlaid with mutant K30R (**A**) and P40L (**B**) with the mutated residue side-chains shown as sticks. RMS values between control LEN and mutant are indicated. Expanded regions highlight key areas of interest in greater detail. Control (C) monomers are shown in pink (monomer A) and blue (monomer B), with mutant (M) structures in green (monomer A) and orange (monomer B). Hydrogen bonds are shown as yellow dashed lines with lengths given, changes in lengths are shown in red text on mutant images. Framework regions (FR) 2 and 3 are shown to help locate positions within the structure.

For LEN K30R, the computational analysis indicates that the conformational changes induced by the conservative substitution of lysine for arginine at position 30, which involves swapping like for like of positively charged basic residues at the surface-exposed CDR1, are quite subtle with an overall root mean square (RMS) value of 0.057 Å (Fig. 6A). This is not surprising given that this position has been reported to tolerate substitution well without much impact on protein structure, as observed for a serine to asparagine mutation at the same site (S30N) in AL-09, an amyloidogenic member of the κI O18:O8 germline^41^. As seen in Fig. 6A the only detectable structural changes include minor alterations in hydrogen bond length between residues Y25 and S29 and the addition of a hydrogen bond of 2 Å between the long flexible arginine side-chain and adjacent backbone carbonyl of S29. These findings are in good agreement with the experimentally determined minimal alterations in protein conformational dynamics and thermodynamic stability (Fig. 5F).

In earlier work mutation of P40, a residue residing in a β-turn within the FR2 loop between β-strands D and E, to leucine was demonstrated to have profound impact on the solubility and aggregation properties of LEN, and was able to promote fibril formation under physiological conditions without the need for addition of preformed misfolded protein seeds (unlike the Q89H and Y96Q LEN mutants)^17^. Proline at position 40 is also present and highly conserved in 98% of all κ and λ germline sequences, further highlighting the importance of this residue^17, 42^. As noted above, however, the ^15^N CPMG relaxation dispersion data for the P40L mutant of LEN (Figs 4H and 5G) are unequivocal, and clearly not consistent with the suggestion that the proline to leucine substitution at this position increases the protein backbone mobility in the FR2 loop relative to LEN^10, 17^. The computational analysis yielded a low RMS value of 0.023 Å, confirming the lack of increased dynamics in the FR2 loop (Fig. 6B). The previous study also proposed that the P40L mutation facilitates interactions between residues of the neighboring FR3 region, specifically by displacing interchain interactions between Q38 residues across the dimer interface, and interrupting the K39-Q81 and R61-D82 hydrogen bonding^17^. To investigate this possibility, we used computational analysis to show that these inter and intrachain hydrogen bonds are not perturbed by the P40L substitution (Fig. 6B). Interestingly, analysis of the dimer contacts using PISA revealed that residue 40 is the only one among the eight SMA-like mutations which resides outside of the dimer interface in LEN, but becomes incorporated into the dimer interface following the proline to leucine substitution (Supplementary Fig. S8). This may be rationalized by the highly hydrophobic leucine residue (3.8 based on the Kyte and Doolittle scale)^43^ preferring to be within a buried interface rather than surface exposed.

**Figure 7:**
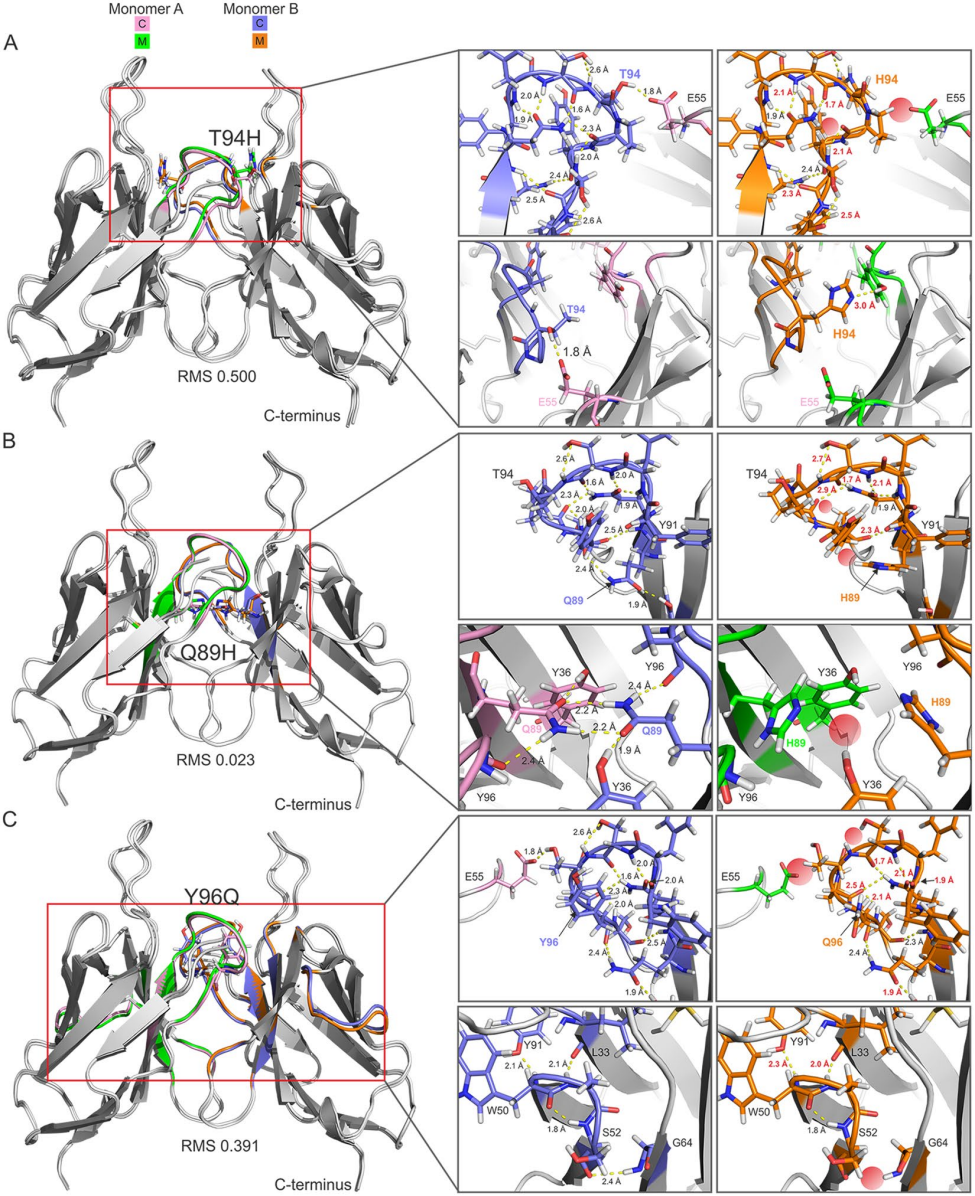
Computational analysis of mutation-induced structural changes for LEN T94H, Q89H and Y96Q. Dimer structure of native LEN is shown as a cartoon overlaid with mutant T94H (**A**), Q89H (**B**) and Y96Q (**C**) with the mutated residue side-chains shown as sticks. RMS values between control LEN and mutant are indicated. Expanded regions highlight key areas of interest in greater detail. Control (C) monomers are shown in pink (monomer A) and blue (monomer B), with mutant (M) structures in green (monomer A) and orange (monomer B). Hydrogen bonds are shown as yellow dashed lines with lengths given; changes in lengths are shown in red text on mutant images. Red spheres indicate loss of hydrogen bond.

Next, we analyzed the Q89H, T94H and Y96Q mutations that displayed significant differences in the ^15^N-^1^H HSQC NMR spectra and conformational exchange dynamics relative to LEN. The Q89H and Y96Q mutants have stabilities 1.0 and 3.2 kcal/mol lower than LEN, respectively, while the T94H substitution is stabilizing with a ΔΔ*G*_*unf*_ value of −0.7 kcal/mol^10^. To investigate the differences between these mutants we performed an exhaustive analysis of inter and intrachain interactions. For T94H, a number of changes in hydrogen bond length and a loss of hydrogen bonding between backbone and side-chain atoms are observed, consistent with the largest RMS value of 0.5 Å out of all of the mutations studied (Fig. 7A). The most profound alteration is the loss of a hydrogen bond between interfacial residues T94 and E55 on opposite sides of the dimer (Fig. 7A, expanded region, top). However, despite the removal of the T94-E55 hydrogen bond, the increase in stability observed experimentally for the T94H mutant could be attributable to the creation of new intersubunit contacts between the histidine residue at position 94 and Y90 on the adjacent monomer (Fig. 7A, expanded region, bottom and Supplementary Fig. S9). Indeed, the structural restraints imposed by the formation of the H94-Y90 hydrogen bond across the dimer interface (Supplementary Fig. S9) are likely responsible for the quenching of conformational exchange dynamics found for LEN T94H (c.f., Figs 4C and 5B).

Glutamine 89 in LEN forms a network of hydrogen bonds with neighboring residues on the adjacent monomer. For the Q89H mutant of LEN, losses of multiple intra and interchain hydrogen bonds are observed (Fig. 7B, expanded region, bottom). There is also substantial alteration of most of the hydrogen bonds within the CDR3 loop (Fig. 7B, expanded region, top). Interestingly, in spite of the sizeable changes in protein conformational dynamics, the overall RMS value of 0.023 Å was found to be relatively low. For Y96Q, the altered residue-specific dynamics between LEN and the mutant structure extend beyond the CDR3 loop containing the site of mutation (Fig. 7C) resulting in a large RMS of 0.391 Å. Interactions between the CDR2 and CDR3 loops, through a E55-T94 hydrogen bond, are lost upon mutation (Fig. 7C, expanded region, top and Supplementary Fig. S9), which is consistent with both loops having increased flexibility and the excessive exchange broadening observed in response to the Y96Q substitution. Further intramolecular changes, including loss of a hydrogen bond between S52 and G64, are noted for several residues along the β-strands that form the protein core and are shown as colored regions within Fig. 7C.

## Discussion

The conversion of immunoglobulin light-chain variable domains to amyloid is widely believed to occur through partially unfolded protein intermediates made accessible through destabilizing amino acid mutations^9, 10, 14, 42^. For the pathogenic SMA κ4 Ig V_L_, for which no high-resolution structure is available, the determination of residue-specific structural and dynamic information is crucial for gaining an improved understanding of the protein aggregation mechanism that is likely the basis for its cytotoxicity. Our comparative NMR analysis of LEN and SMA Ig V_L_s indicates that, based on the nearly identical backbone ^13^Cα and ^13^Cβ chemical shifts, these two light-chains share a common overall fold for the majority of residues found in the β-rich framework regions. Concurrently, however, at physiological pH SMA was found to display significant differences in protein backbone motions on the millisecond timescale with respect to LEN (c.f., Figs 4 and 5, and Supplementary Fig. S6). On the whole, as a result of the eight amino acid mutations relative to the LEN sequence, SMA exhibits enhanced conformational flexibility suggestive of partial protein unfolding as reflected by the elevated *R*_*ex*_ values and/or considerable chemical exchange broadening for many residues, particularly those located in the CDR1, CDR2 and CDR3 loops; the latter conclusion is also in line with the somewhat elevated rotational correlation time for the SMA dimer relative to LEN estimated from ^15^N longitudinal and transverse spin relaxation data. Additionally, consistent with the findings of earlier low-resolution biophysical measurements^26^, the conformational dynamics of SMA at physiological pH resemble those of LEN at pH 2 (c.f., Fig. 4A and E), where our previous NMR studies^31^ revealed that acid driven destabilization of LEN is accompanied by a ~3–4-fold increase in the population of partially unfolded protein conformers involving residues at the dimer interface.

Previously it has been shown that the effects of SMA-like mutations on the thermodynamic stability of LEN are non-cooperative^10^. In order to evaluate the contribution of each amino acid substitution to shaping the dynamic conformational landscape of SMA, we quantified the slow conformational exchange dynamics for the eight SMA-like point mutants of LEN by ^15^N relaxation dispersion techniques and, based on the results of the NMR studies, performed additional *in silico* analysis for a subset of these proteins. The NMR studies indicate that a combination of only three mutations located within or directly adjacent to the CDR3 loop at the dimer interface (Q89H, T94H and Y96Q) largely accounts for the observed differences in protein backbone dynamics between LEN and SMA. These mutations include two of the most strongly destabilizing mutations (Q89H and Y96Q with ΔΔ*G*_*unf*_ values of 1.0 and 3.2 kcal/mol, respectively; Fig. 5), with Y96Q exhibiting conformational exchange dynamics that are considerably enhanced relative to SMA (Fig. 4D), as well as the stabilizing T94H substitution (ΔΔ*G*_*unf*_ = −0.7 kcal/mol) for which the millisecond timescale protein backbone motions are effectively completely quenched (Fig. 4C). The impact of the remaining five substitutions (S29N, K30R, P40L, S97T and I106L) on the backbone dynamics of LEN was found to be minimal, in spite of the fact that they include the fairly destabilizing P40L (ΔΔ*G*_*unf*_ = 0.7 kcal/mol) and the most stabilizing S29N (ΔΔ*G*_*unf*_ = −1.0 kcal/mol) substitutions. *In silico* analysis of the Q89H, T94H and Y96Q mutations shows that they perturb a consistent set of residues within the CDR3 region surrounding the mutation site and interactions with surrounding loops (Fig. 7 and Supplementary Fig. S9). For Y96Q, all interactions between the CDR3 loop and surrounding regions are abolished through the loss of a hydrogen bond between E55 and T94 residues in adjacent monomers. The Q89H mutation also causes loss of hydrogen bonding at the base of this loop (at the termini of FR3 and FR4 regions, strands H and I respectively) (Supplementary Fig. S9). On the other hand, while the E55-T94 hydrogen bond is similarly lost for the T94H mutant, a new hydrogen bond which re-stabilizes the CDR3 loop is formed between H94 and Y90 on the adjacent monomer (Supplementary Fig. S9). Taken together, these findings suggest that the CDR3 loop is key to regulating the thermodynamic stability and amyloidogenicity of these light chains—for the Q89H and Y96Q substitutions, disrupted hydrogen bonds appear to contribute to the observed decreased thermodynamic stability (and ability to form amyloid fibrils under seeded conditions)^17^, whereas, for LEN T94H the key hydrogen bond that is lost is replaced with another one resulting in increased protein stability and reduced amyloidogenicity. Interestingly, while previous studies have pointed to residue L40 as being an important determinant of amyloid formation by SMA *in vitro* ^10, 17^, we did not find any evidence for significant changes in either the protein backbone structure or dynamics due to the P40L mutation in LEN. Based on the computational analysis, however, we do note some alteration in the residues involved in the dimer interface (Supplementary Fig. S8), which may be sufficient to alter the protein solubility and provide the necessary driving force to allow the protein access to the amyloidogenic landscape under the experimental conditions employed in the earlier work (pH 7.5 with agitation)^17^. We hypothesize that the presence of two additional leucine residues within the dimer cavity may not affect the stability of the dimer due to being sequestered within the hydrophobic cavity. In contrast, in the monomeric state the additional leucine residue will be solvent exposed and affect the protein stability. It is therefore worth noting that previous studies on P40L were performed at low protein concentrations, where the fraction of monomer is higher compared to the predominant dimer population present in the current study.

Our findings provide a molecular level rationale for and reinforce the observations of the study by Davis *et* *al*.^18^, which concluded that mutation of residues H89, H94 and Q96 is sufficient for κ4 light-chains to form amyloid *in vivo* by a gain-of-function phenomenon. At the same time, given that SMA is highly amyloidogenic but that, in the absence of seeding, neither of the most strongly destabilizing Q89H and Y96Q mutants of LEN assemble efficiently into fibrils forming mostly nonfibrillar aggregates instead^17^ and that another highly destabilized κ4 Ig V_L_ did not form amyloid while its more stable variants were found to do so^10^, they also highlight the rather complex and nuanced nature of the correlations between Ig V_L_ conformational flexibility, thermodynamic stability and amyloidogenicity. Notably, our sequence-specific NMR relaxation dispersion data show that residues located outside the three dynamic hot-spot regions are relatively immobile in both LEN and SMA, in contrast to Y96Q and, to some extent, Q89H mutants of LEN, which exhibit intermittent elevated backbone dynamics outside these regions, including β-strands D and G. In the case of LEN Y96Q, by perturbing its β-sheet structure, the mutation apparently renders the protein too unstable to readily form fibrils without seeding, and, consequently, it is unlikely that an even more thermodynamically unstable combination of Q89H and Y96Q substitutions in LEN would result in an Ig V_L_ capable of forming fibrils efficiently. These observations underscore the critical role played by appropriately positioned stabilizing substitutions, such as T94H in the CDR3 loop for SMA, which restore key protein interactions at those sites and in combination with the destabilizing mutations provide the correct amount of net destabilization required to form unfolded intermediates that ultimately lead to amyloid assembly. Taken together with the recent reports by Ramirez-Alvarado and co-workers^44, 45^, where a combination of stabilizing and destabilizing mutations was found to be key for conferring amyloidogenicity to several κ1 Ig V_L_ sequences, our findings are likely applicable to other immunoglobulin light-chains and, indeed, to other amyloid-forming proteins.

## Methods

### NMR Spectroscopy

The LEN and SMA plasmids^7^ were kind gifts from Prof. Fred J. Stevens (Argonne National Laboratory). Eight additional plasmids, each corresponding to a single SMA-like point mutation (S29N, K30R, P40L, Q89H, T94H, Y96Q, S97T or I106L) in LEN, were constructed by using the LEN plasmid DNA, appropriate primers and the QuikChange site-directed mutagenesis protocol (Stratagene). All ^15^N and ^13^C, ^15^N labeled proteins were overexpressed in *E*. *coli* C41 (DE3) cells as described in detail previously^30, 31^. Samples used for the NMR measurements consisted of proteins at concentrations ranging between ~1–2 mM in aqueous solution containing 20 mM sodium phosphate, 100 mM NaCl, 7% (v/v) D_2_O and 0.02% (w/v) sodium azide at pH 6.5 in Shigemi microcells.

NMR experiments were performed at 25 °C on Bruker DRX-600 and DRX-800 spectrometers equipped with cryo-probes having z-axis gradients. The sequential backbone resonance assignments were obtained by using ^13^C, ^15^N-labeled proteins and 3D HNCA, HN(CO)CA and/or HN(CA)CB experiments^46^. The ^15^N relaxation and relaxation dispersion measurements designed to probe the fast and slow timescale protein backbone dynamics were carried out using the pulse schemes of Kay and co-workers^47, 48^, exactly as described in our previous study of LEN^31^. Spectra were processed in NMRPipe^49^ and the sequential resonance assignments were made using Sparky^50^. The analysis of ^15^N relaxation data was performed as described in detail in our earlier study^31^, with the following minor modifications. The residue-specific relaxation dispersion trajectories at 14.1 and 18.8 T were first fit within the *CATIA* program^51^ kindly provided by Prof. Lewis E. Kay (University of Toronto). For each calculated trajectory the chemical exchange contribution to ^15^N transverse relaxation (*R*_*ex*_) was obtained as the difference in *R*_2,*eff*_ values at the lowest (33.3 Hz) and highest (1000 Hz) CPMG frequencies, with the *R*_*ex*_ value of 3 Hz at 14.1 T used as a cut-off to identify the residues exhibiting significant conformational exchange dynamics. As described in detail in the Results section, in analogy to LEN^31^ for all the κ4 Ig V_L_s investigated in the present study the elevated conformational dynamics were found to be largely clustered in three distinct protein regions, which were identified by inspection of the *R*_*ex*_ vs. residue number plots. For each region the relaxation dispersion trajectories were simultaneously fit to the two-site exchange model^40^, where possible, to determine the global dynamic parameters including the exchange rate and population of minor conformer.

### Circular Dichroism Measurements

Near-UV CD spectra were recorded from 320 to 250 nm for solutions of LEN, SMA, and the eight SMA-like mutants of LEN mutants at pH 6.5 and 25 °C using a JASCO J-815 spectrometer, a rectangular cuvette with 10 mm path length and protein concentrations ranging from 0.05 to 3.0 mg/mL. For each Ig V_L_ protein, the association constant (*K*_*a*_) was estimated using the method of Azuma *et al*.^38^, previously applied to LEN by Fink and co-workers^24^. Namely, *K*_*a*_ as well as the ellipticities of the monomeric ([Θ]_*m*_) and dimeric ([Θ]_*d*_) forms were extracted within the GraphPad Prism software by non-linear curve fitting of the observed ellipticity at 282.5 nm ([Θ]_*obs*_) as a function of total protein concentration (*C*_*T*_) according to^24, 38^:

$${[{\rm{\Theta }}]}_{obs}={[{\rm{\Theta }}]}_{m}{C}_{m}/{C}_{T}+{[{\rm{\Theta }}]}_{d}{C}_{d}/{C}_{T}$$

and

$${K}_{a}=\frac{1}{2}\cdot M{C}_{d}/{({C}_{m})}^{2},$$

where *C*_*m*_ and *C*_*d*_ are the monomer and dimer concentrations, respectively, and *M* is the molecular weight of the monomer.

### *In Silico* Analysis

LEN mutants K30R, P40L, T94H, Q89H and Y96Q were generated using the crystal structure of LEN (PDB entry 1LVE) as a template structure. Residues were altered *in silico* using the PyMOL mutagenesis wizard^52^ and subjected to energy minimization in full atom mode, while retaining backbone and side-chain symmetry of the dimer throughout the simulation using the macromolecular modelling suite Rosetta. For validation, dimer symmetry was checked by matching the coordinates of randomly chosen residues of chains A and B. One hundred structures were generated for each mutation and superimposed using a maximum likelihood method as part of the program THESEUS^53^. These were visualized in PyMol to check that models preserved conformational sampling density and all converged to a similar end point. The lowest scoring model for each mutation based on the Rosetta scoring function, which represents the most optimal model and most likely native structure, was taken for further analysis. To ensure that any changes observed between the starting and mutant structures were not a result of relaxation away from crystal contacts and a true outcome, which arises from the substituted amino acid, the template was also subjected to the same minimization procedure. The protein interfaces, surfaces and assemblies service (PISA) program^54^ was used to investigate changes in the interfacial contacts between mutants and starting structure. All protein structure rendering was performed using PyMol.

### Data Availability

The datasets generated during and/or analyzed during the current study are available from the corresponding author on reasonable request.

## Electronic supplementary material

Supplementary Information
