# Supplementary material for: Effect of amino acid mutations on the conformational dynamics of amyloidogenic immunoglobulin light-chains: A combined NMR and in silico study
Source: Sci Rep. 2017 Sep 4;7:10339. doi: 10.1038/s41598-017-10906-w (PMC5583243; doi:10.1038/s41598-017-10906-w)
Supplement: Supplementary file 1 — Supplementary Information [file 41598_2017_10906_MOESM1_ESM.pdf]

Supplementary Information

Effect of amino acid mutations on the conformational dynamics of amyloidogenic immunoglobulin light-chains: A combined NMR and *in silico* study

Sujoy Mukherjee[1,2], *Simon P. Pondaven*[1], *Kieran Hand*[3], *Jillian Madine*[3], *Christopher P. Jaroniec*[1,*]

[1]Department of Chemistry and Biochemistry, The Ohio State University, Columbus, Ohio 43210, USA

[2]Structural Biology and Bio-Informatics Division, Indian Institute of Chemical Biology, Kolkata, India 700032

[3]Institute of Integrative Biology, University of Liverpool, UK

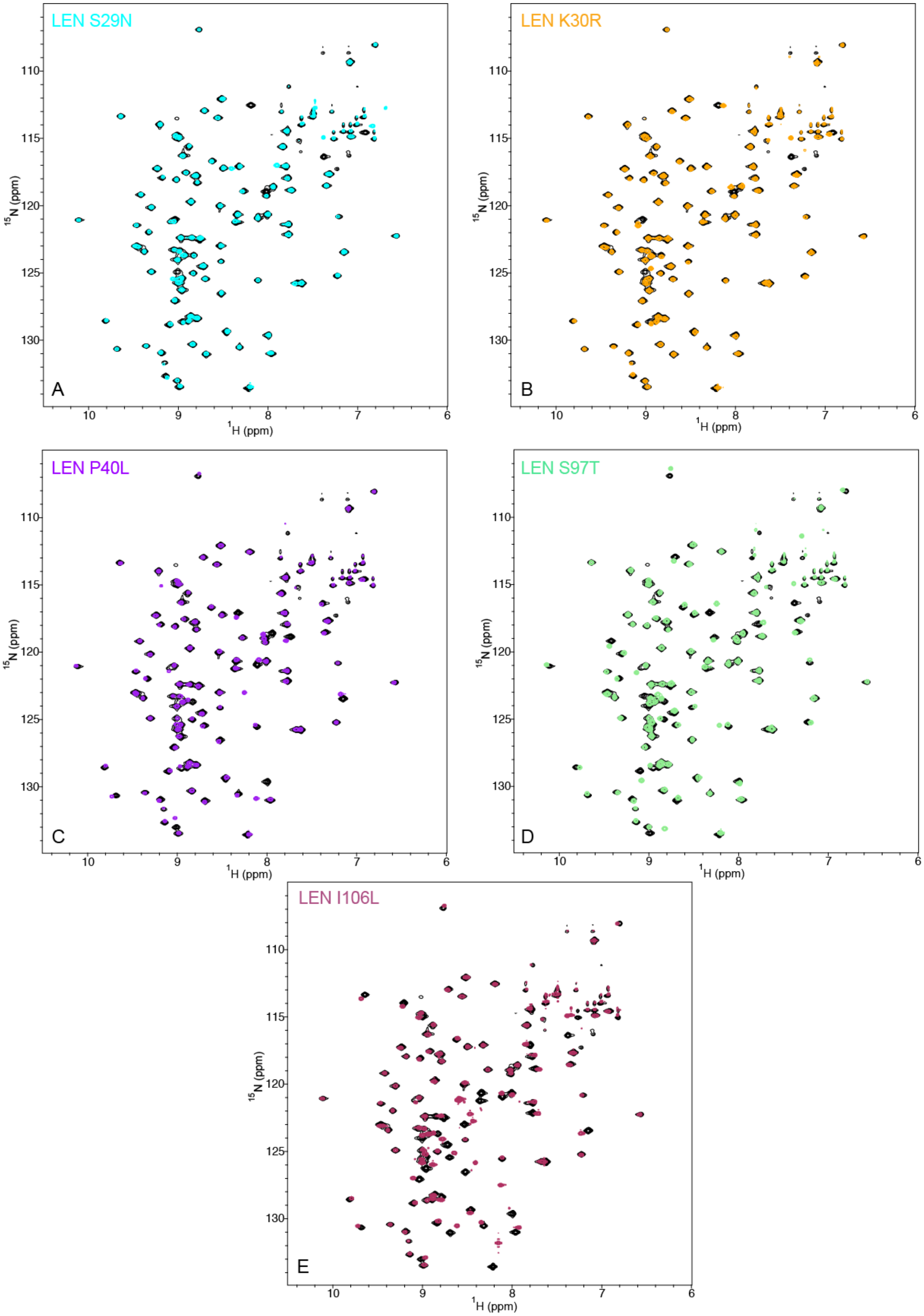

Figure S1. Two dimensional ^{15}N-^{1}H HSQC spectra of ^{15}N-labeled (A) LEN S29N, (B) LEN K30R, (C) LEN P40L, (D) LEN S97T and (E) LEN I106L recorded at 800 MHz ^{1}H frequency and 25 ^{o}C, overlaid on the spectrum of LEN (black contours).

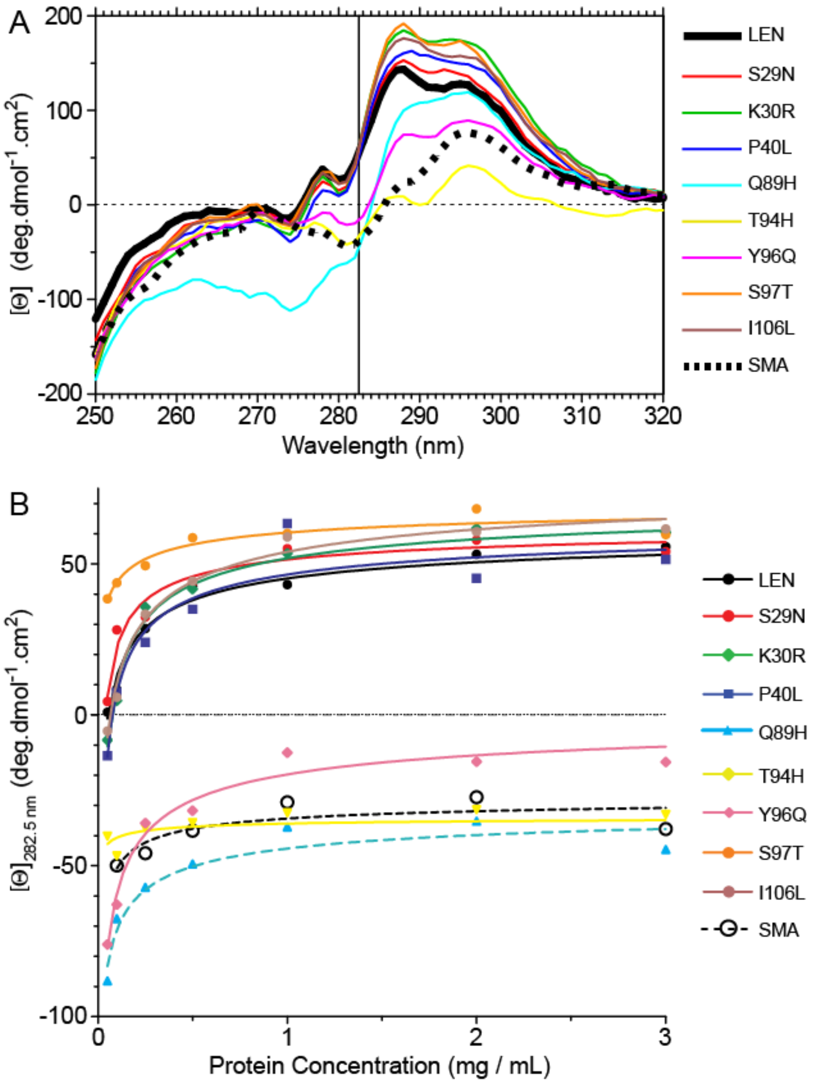

Figure S2. (A) Near-UV CD spectra of LEN, SMA and the eight SMA-like mutants of LEN at pH 6.5 (see legend) recorded for protein solutions with concentration of 3 mg/mL. The wavelength of 282.5 nm is denoted by the vertical line. (B) Plots of the ellipticity at 282.5 nm ($\Theta_{282.5\ nm}$) against protein concentration for LEN, SMA and the eight SMA-like mutants of LEN (see legend), with lines of the corresponding color showing the best non-linear fits used to determine the association constants as described in the Methods section (c.f., Supplementary Table S3). The non-linear fitting routine did not converge for LEN Q89H; for reference, a curve calculated using the association constant for LEN is shown as a dashed cyan line.

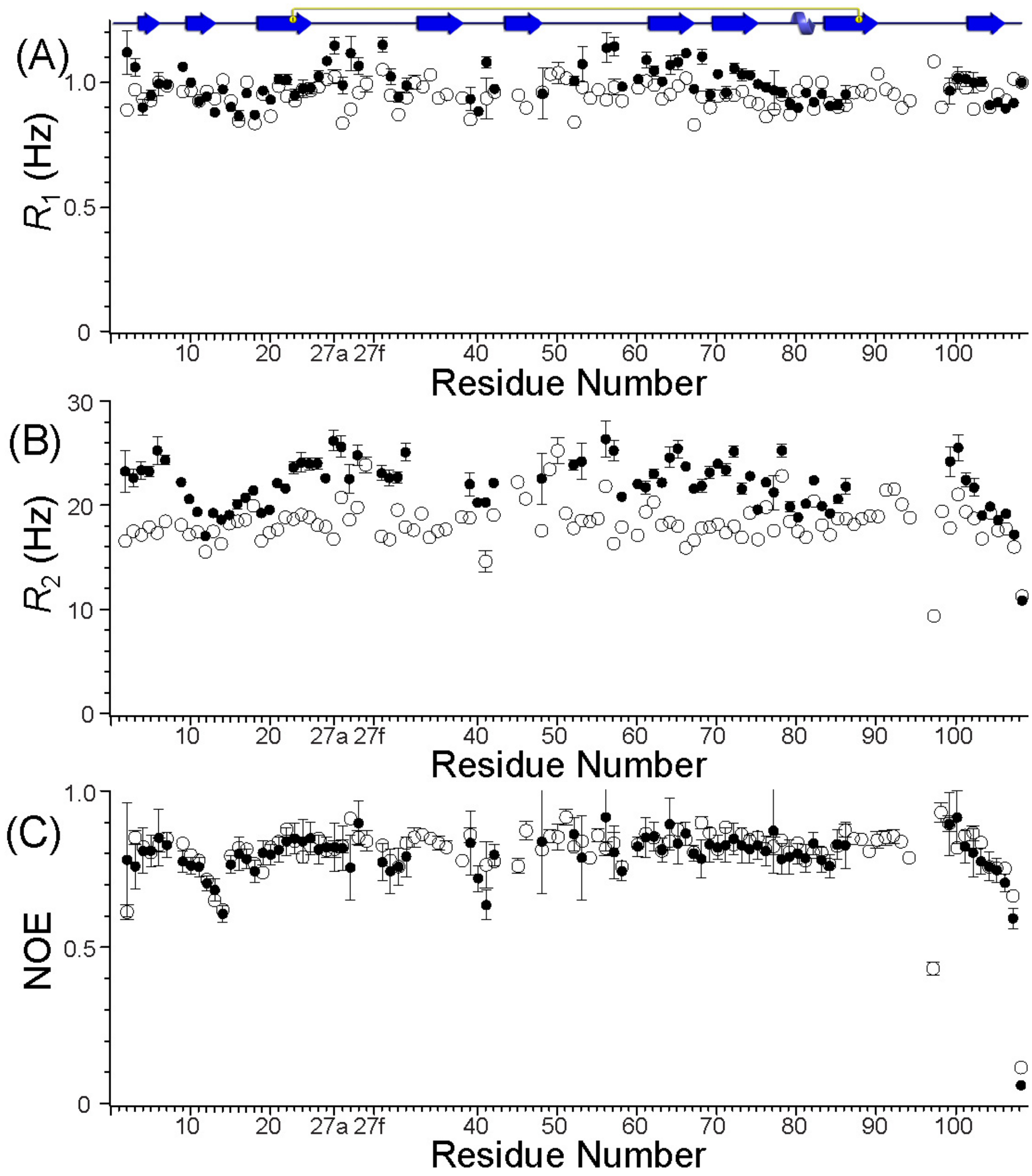

Figure S3. Backbone amide ^{15}N spin relaxation measurements in SMA used to characterize the fast picosecond-nanosecond timescale motions. Plots of (A) ^{15}N R_1, (B) ^{15}N R_2 and (C) $\{^1H\}$-^{15}N heteronuclear NOE (filled circles) are shown as a function of the residue number. The spin relaxation measurements were performed as described in Farrow *et. al.* [1] at 600 MHz ^{1}H frequency, 25˚C, and pH 6.5, and the relaxation rates extracted by fitting the trajectories to decaying single exponentials. For comparison, the spin relaxation rates for LEN, obtained as part of an earlier study [2], are shown as hollow circles. The secondary structure of LEN [3] is indicated above the top panel.

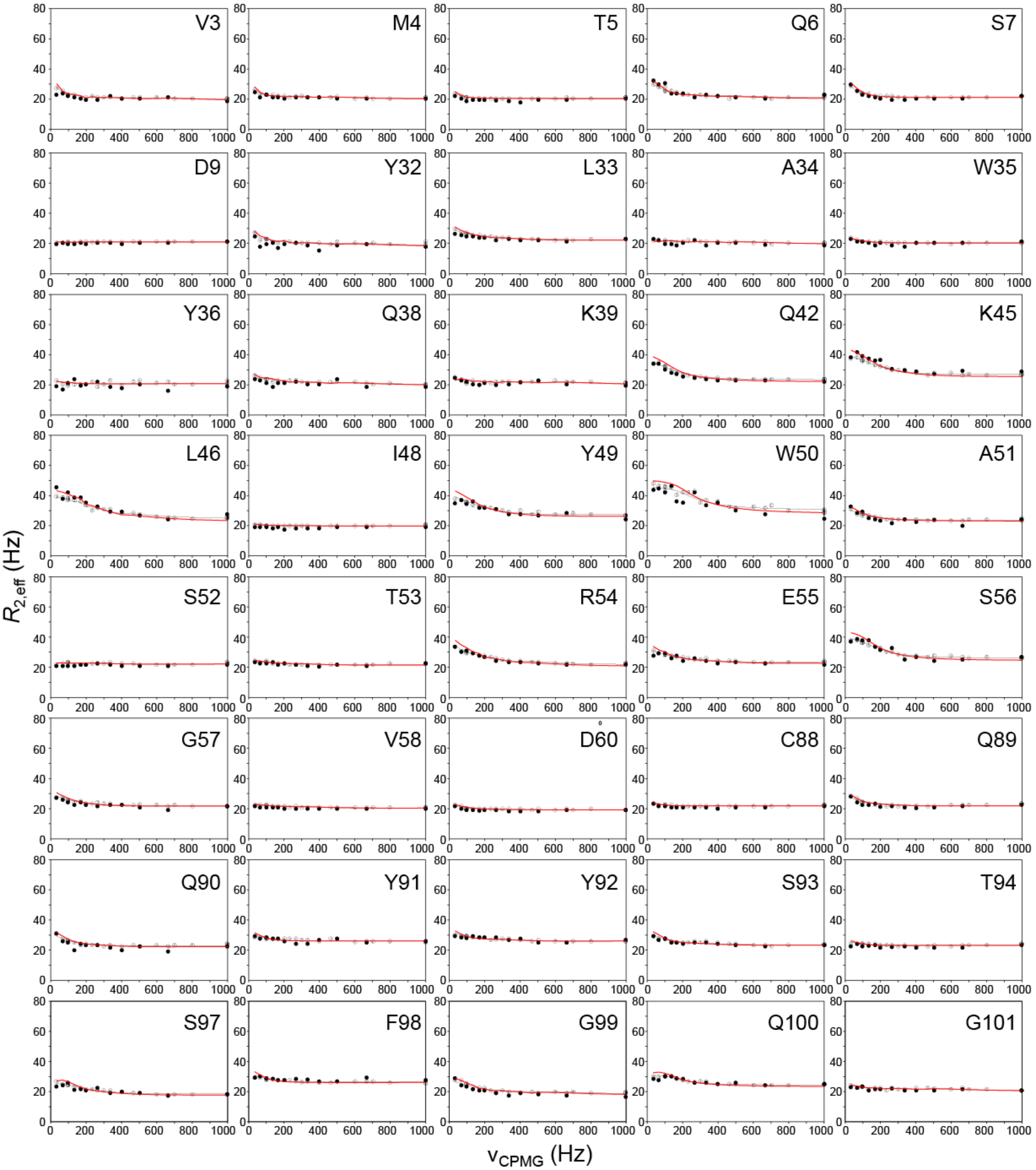

Figure S4. ^{15}N CPMG relaxation dispersion trajectories for residues from the three dynamic regions of the most thermodynamically stable mutant of LEN (S29N) at pH 6.5 (see main text for details). Experiments were performed at 800 MHz ^{1}H frequency for two different protein concentrations, 0.9 mM (filled circles) and 1.9 mM (hollow circles). For reference, fits of CPMG trajectories for the 1.9 mM protein sample are shown as black lines, and for the 0.9 mM sample such fits yield minor conformer populations, p_B, of 9.5 ± 1.9 %, 3.2 ± 0.1 %, and 3.5 ± 0.4 % for dynamic regions 1, 2 and 3, respectively, that are in good agreement with the 9.0 ± 1.9 %, 3.2 ± 0.1 %, and 3.5 ± 0.4 % values

obtained for the 1.9 mM sample (c.f., Supplementary Table S4). Also shown as red lines are calculated trajectories corresponding to minor conformer populations increased by 45 % (corresponding to the roughly two-fold protein sample dilution). If the relaxation dispersions were caused by exchange processes between the monomeric and dimeric forms of the protein as opposed to dynamics within the protein dimer, the experimental CPMG trajectories would be expected to follow these calculated trajectories. Similar data for LEN Y96Q are shown in Supplementary Fig. S5, and the results for both LEN mutants are collectively discussed in the Supplementary Fig. S5 caption.

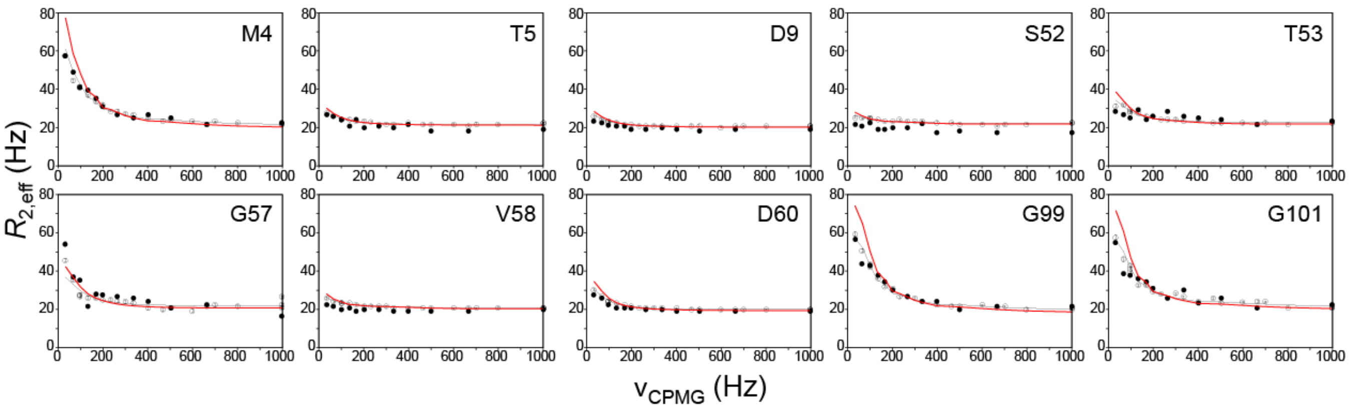

Figure S5. Same as Supplementary Fig. S4 but for the least thermodynamically stable mutant of LEN (Y96Q) at concentrations of 0.7 mM (filled circles) and 1.4 mM (hollow circles). For reference, fits of CPMG trajectories for the 1.4 mM protein sample are shown as black lines, and for the 0.7 mM sample such fits yield minor conformer populations, p_B, of 14.2 ± 0.2 % and 3.7 ± 0.1% for dynamic regions 1 and 2, respectively, that are in reasonable agreement with the 17.0 ± 0.4 % and 6.8 ± 0.3 % values obtained for the 1.4 mM sample (c.f., Supplementary Table S4). Also shown as red lines are calculated trajectories corresponding to minor conformer populations increased by 45% (corresponding to the two-fold protein sample dilution). Altogether, the similarity between the residue-specific trajectories irrespective of protein concentration shown in Supplementary Fig. S4 and S5 for the LEN S29N and Y96Q mutants suggests that these data report primarily on conformational dynamics within the protein dimers and are not significantly affected by monomer-dimer exchange phenomena. This conclusion is supported by the fact that the experimental CPMG trajectories for the dilute protein samples clearly do not follow the red curves at low ν_{CPMG} values, where the trajectories are most sensitive to exchange phenomena, for residues exhibiting the largest relaxation dispersions (e.g., Y49 and R54 for LEN S29N and M4, G99 and G101 for LEN Y96Q) – if the experimentally observed relaxation dispersions were caused by exchange between monomeric and dimeric forms of the proteins as opposed to dynamics within the protein dimers (i.e., if the minor state in exchange with the major dimer state corresponded to the monomer) the experimental CPMG trajectories for the dilute protein samples would be expected to follow the calculated trajectories shown as red lines. In addition, the fact that the relaxation dispersion profiles for the different Ig V_Ls (Fig. 4 of the main text) are uncorrelated with the measured dimer association constants (Supplementary Table S3) while exhibiting a reasonable correlation with the $\Delta\Delta G_{unf}$ values (Supplementary Fig. S7), further underscores the conclusion that the CPMG experiments report on the conformational dynamics within Ig V_L dimers.

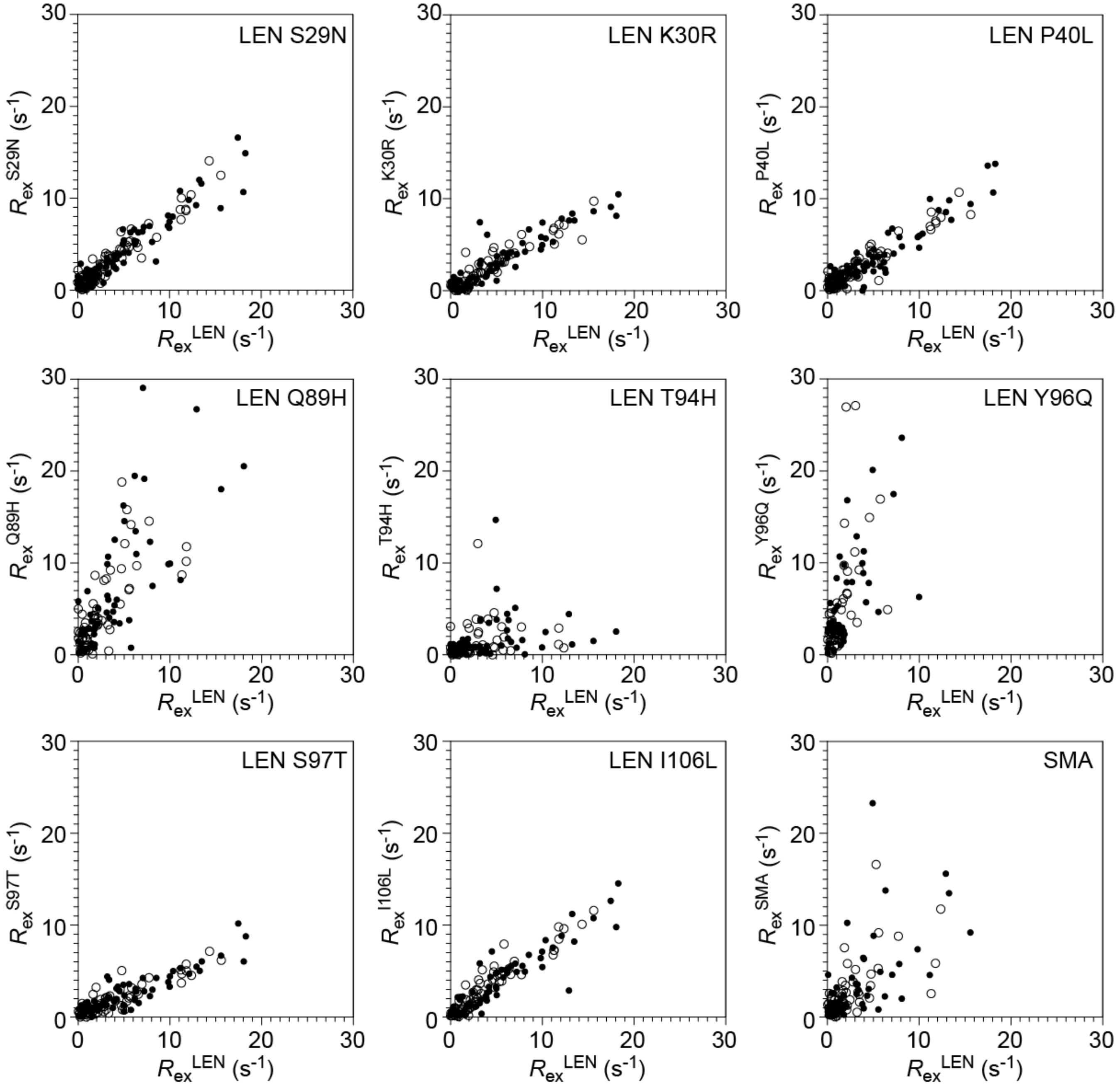

Figure S6. Plots of residue-specific chemical exchange contributions to ^{15}N transverse relaxation, R_{ex}, at 600 MHz (open circles) and 800 MHz (filled circles) ^{1}H frequency for LEN versus SMA and S29N, K30R, P40L, Q89H, T94H, Y96Q, S97T and I106L mutants of LEN as indicated in the insets (see Fig. 4 of the main text for plots of R_{ex} as a function of residue number). These correlation plots show that SMA and Q89H, T94H and Y96Q mutants display the largest differences in conformational dynamics relative to LEN.

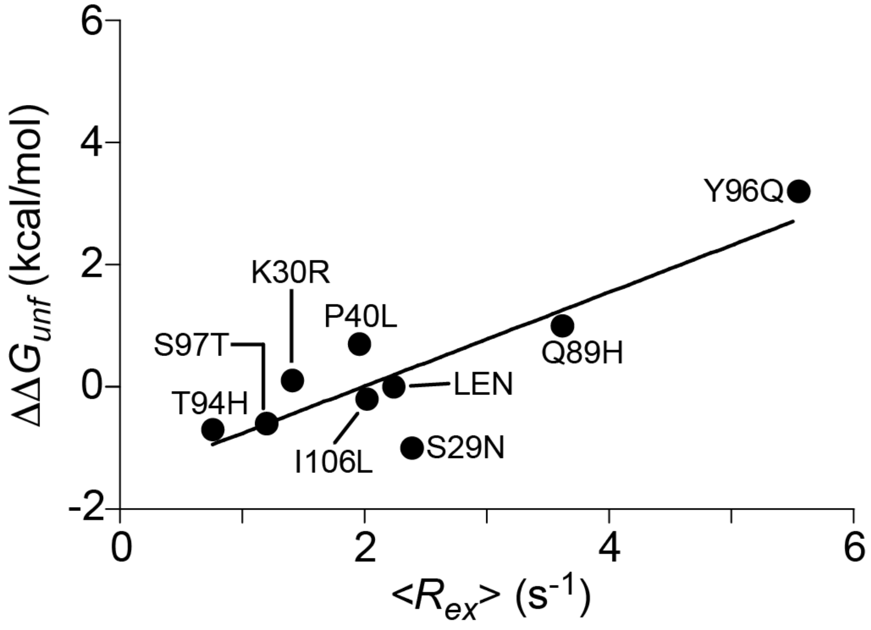

Figure S7. Correlation between millisecond timescale protein backbone conformational flexibility as represented by the average residue-specific chemical exchange broadening at 600 MHz ^{1}H frequency, $\langle R_{ex} \rangle$, and relative thermodynamic stability of the protein with respect to LEN, $\Delta\Delta G_{unf}$ (see Fig. 5 and main text for additional details). A linear fit of the data ($R^2 = 0.77$) is also shown.

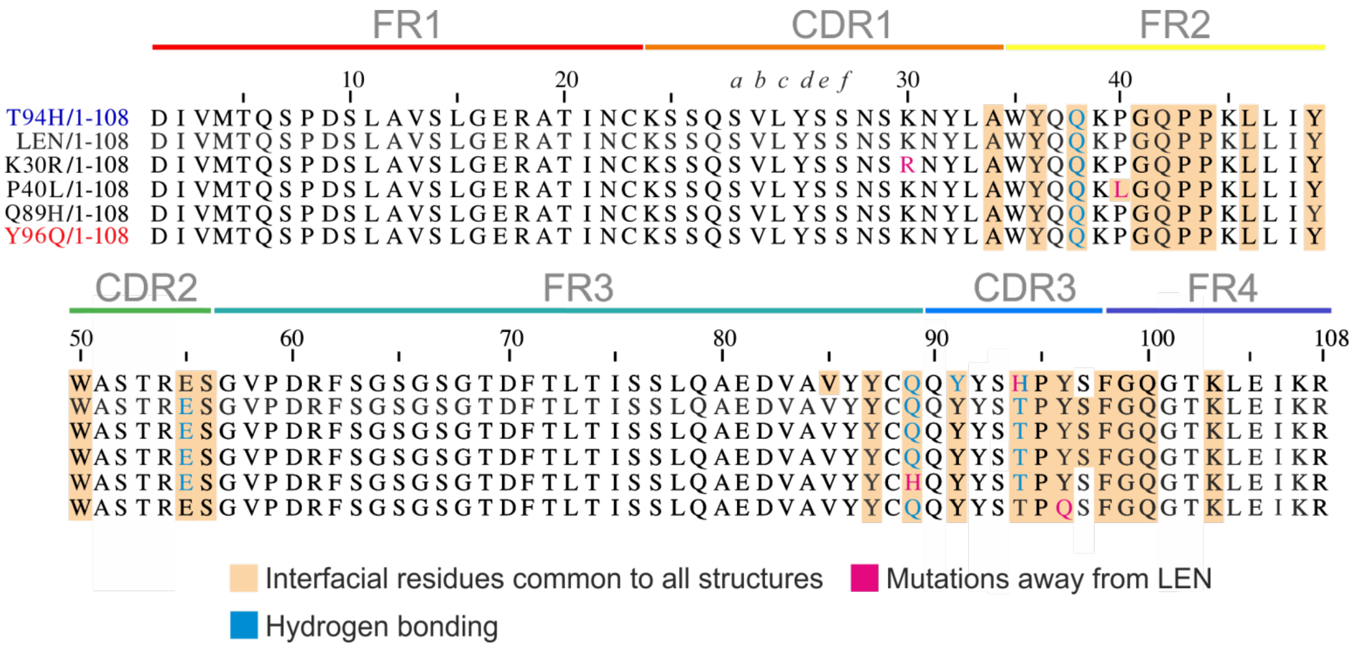

Figure S8. Combination of PISA and comparative analysis between mutated and control structures processed under identical conditions. Mutants highlighted in blue (T94H) and red (Y96Q) indicate the most and least thermodynamically stable ones from among those considered. Residue and region annotation were taken from Stevens and co-workers [4] and are presented according to Kabat nomenclature [5]. In the case where N-terminal residue D1 was detected to be part of the interface, it was omitted from analysis, as previously done by Baden et al [6].

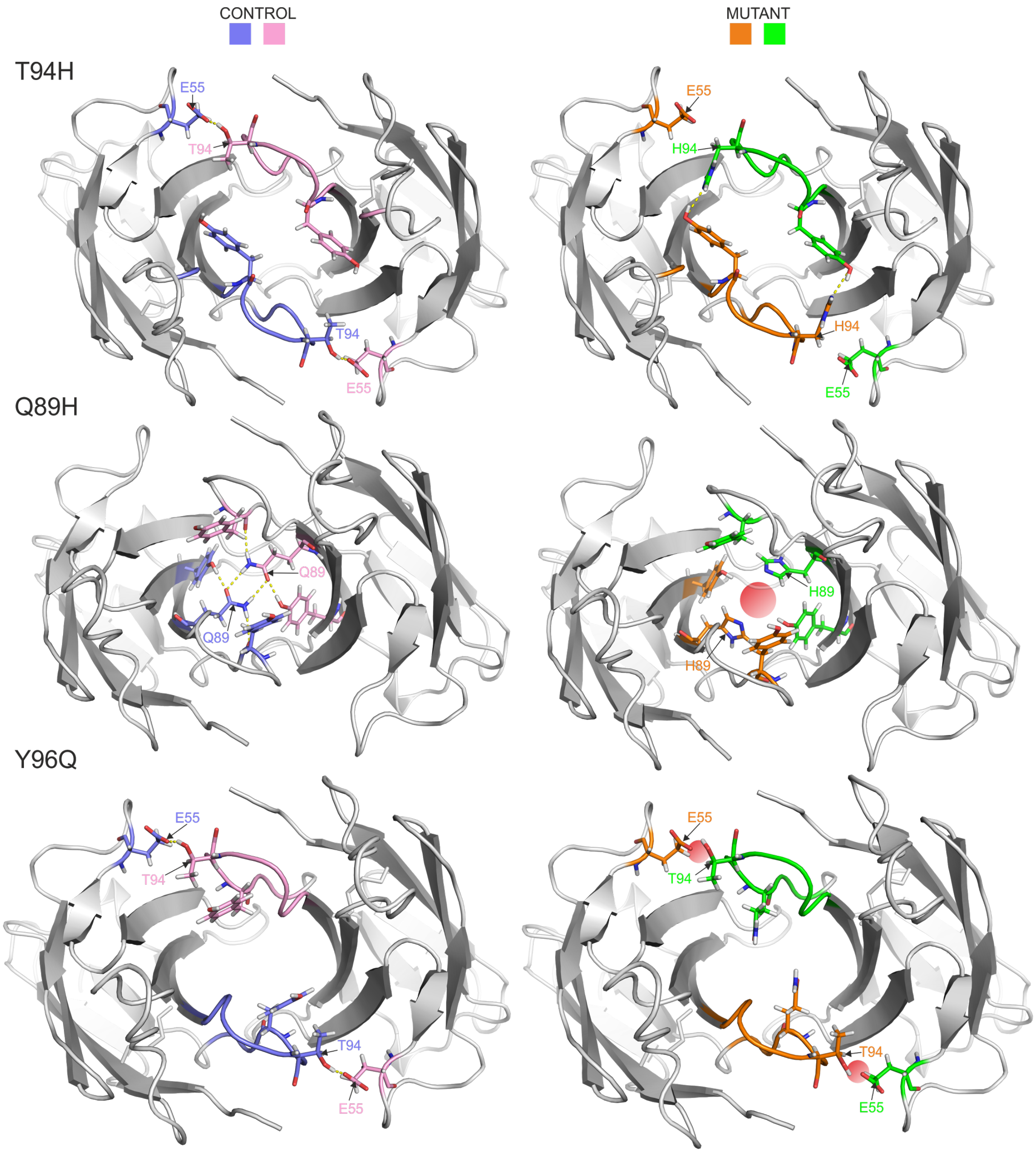

Figure S9. Disruption of key interface residues determined by computational analysis of mutation-induced structural changes for T94H, Q89H and Y96Q mutants of LEN. Dimer structure of native LEN is shown as a cartoon overlaid with mutant T94H, Q89H and Y96Q, with key side-chains shown as sticks. Control monomers are shown in pink (monomer A) and blue (monomer B) (left), with mutant structures in green (monomer A) and orange (monomer B) (right). Hydrogen bonds are shown as yellow dashed lines, with red spheres used to indicate loss of a hydrogen bond.

Table S1. $^{13}C\alpha$, $^{13}C\beta$, $^{1}H^{N}$ and ^{15}N chemical shifts for SMA at 25 °C and pH 6.5.

Residue Number	δCα (ppm)	δCβ (ppm)	δH^N (ppm)	δN (ppm)
D1	53.339			
I2	62.894		9.034	122.486
V3	62.248		8.742	131.266
M4	52.013		8.984	127.967
T5	61.745	70.676	9.426	118.814
Q6	54.618		10.025	128.212
S7	56.076	65.002	8.696	115.848
P8	63.145	34.909		
D9	57.055	41.041	8.836	118.620
S10	56.941	65.040	7.772	113.123
L11	54.761	45.996	8.847	124.893
A12	50.217	19.681	8.439	128.295
V13	59.836	35.700	8.485	120.287
S14	58.429	64.234	8.614	120.560
L15	56.773	41.549	8.395	121.607
G16	45.156		9.625	112.589
E17	55.863	30.825	7.706	121.027
R18	54.977	32.094	7.994	119.737
A19	50.385	23.082	8.788	127.460
T20	61.369	70.952	7.822	115.706
I21	60.999	41.065	8.940	124.312
N22	53.072	42.668	9.010	123.034
C23	56.013	48.266	9.496	121.902
K24	53.817	36.410	9.118	129.442
S25	56.032	67.032	9.306	119.326
S26	60.681	62.578	8.443	115.785
Q27	53.701	33.001	7.278	116.519
S27a	58.152	62.739	8.679	116.800
V27b	59.381	30.423	8.196	117.582
L27c	54.385		7.571	124.112
Y27d	55.255		9.133	131.522
S27e				
S27f				
N28	51.674	38.982	6.834	112.896
N29	54.640	38.031	8.300	115.973
R30	54.985		7.868	115.467
N31	53.055		9.074	120.768
Y32				
L33				
A34				
W35				
Y36				
Q37				
Q38				
K39	56.020		9.004	131.719
L40	57.190	41.689	8.053	121.512
G41	45.799		8.794	114.580
Q42	53.479	29.817	8.077	120.115
P43				
P44				
K45				

L46				
L47				
I48			7.271	117.117
Y49				
W50				
A51	57.831			
S52	57.964	66.312	8.746	112.152
T53	63.454		9.155	123.264
R54				
E55	53.441			
S56	60.240		8.753	125.863
G57	45.426		8.862	113.814
V58	60.416	32.906	7.610	124.530
P59	63.438	33.619		
D60	55.866	39.712	8.516	119.010
R61	56.818	29.399	7.043	113.667
F62	58.057	40.349	7.738	119.978
S63	57.232	65.440	8.883	114.839
G64	44.010		9.160	112.741
S65	57.083	66.202	9.215	116.053
G66	44.771		8.486	110.850
S67	57.586	64.285	7.067	108.242
G68	48.642		9.025	113.758
T69	60.465	71.451	7.778	114.284
D70	54.145	43.721	6.543	121.055
F71	57.505	43.032	8.920	121.426
T72	61.513	73.230	8.814	116.710
L73	53.222	41.000	8.827	129.211
T74	61.029	70.455	8.910	122.461
I75	59.936	39.121	8.844	126.943
S76	60.256	63.027	8.832	121.191
S77	56.549	62.505	6.694	113.263
L78	57.746	42.566	8.458	125.228
Q79	53.368	31.194	9.019	125.729
A80	56.047	17.581	8.947	124.577
E81	57.476	28.634	8.875	113.512
D82	54.445	41.054	8.048	119.276
V83	65.480	31.471	7.138	122.108
A84	51.573	20.672	8.021	130.025
V85	61.402	32.633	7.644	118.046
Y86	56.637		8.555	123.314
Y87				
C88				
H89				
Q90				
Y91				
Y92				
S93				
H94				
P95				
Q96				
T97				
F98	57.221			
G99	44.937		8.800	107.143
Q100	57.653		8.581	113.857

G101	45.358		6.982	107.254
T102	61.711	72.530	8.025	118.363
K103	56.479	33.319	8.317	128.898
L104	54.560	44.209	8.642	129.921
E105	54.038	33.812	8.783	123.261
L106	53.970	42.781	8.413	120.497
K107	56.298	33.384	8.029	126.202
R108	57.629	31.436	8.128	130.798

Table S2. ^{13}Cα, $^{1}H^{N}$ and ^{15}N chemical shifts for LEN Q89H, LEN T94H and LEN Y96Q at 25 °C and pH 6.5.

Residue Number	δCα (ppm)	δH^N (ppm)	δN (ppm)	δCα (ppm)	δH^N (ppm)	δN (ppm)	δCα (ppm)	δH^N (ppm)	δN (ppm)
	LEN Q89H			LEN T94H			LEN Y96Q		
D1	53.751			53.617					
I2	63.130	9.187	123.735	62.983	9.298	123.700			
V3	62.116	8.971	133.411	62.030	9.012	133.462			
M4	52.279	9.114	129.066	51.907	9.104	128.566	51.93	9.044	128.7
T5	61.995	9.424	119.555	61.848	9.484	119.528	61.76	9.405	119.5
Q6	54.776	9.811	128.425	54.688	9.925	129.453		9.677	129.4
S7	56.323	8.665	116.693						
P8	63.433			63.148			63.08		
D9	57.248	8.860	119.723	57.090	8.892	119.970	56.91	8.844	119.6
S10	57.218	7.793	114.454	56.952	7.819	114.542	56.95	7.764	114.4
L11	54.985	8.983	126.225	54.706	8.945	126.561	54.67	8.898	126.1
A12	50.477	8.464	129.250	50.193	8.464	129.436	50.22	8.449	129.3
V13	59.964	8.355	121.398	59.775	8.363	121.392	59.69	8.309	121.2
S14	59.331	8.767	122.665	59.117	8.774	122.653	59.07	8.738	122.5
L15	57.192	8.531	122.978	56.995	8.540	123.064	56.91	8.504	122.9
G16	45.339	9.640	113.346	45.106	9.631	113.394	45.09	9.607	113.2
E17	55.896	7.763	122.137	55.667	7.791	122.168	55.63	7.735	122.0
R18	55.332	8.018	120.715	55.209	8.053	120.944	55.10	7.973	120.5
A19	50.551	8.796	128.445	50.326	8.793	128.526	50.27	8.781	128.3
T20	61.751	7.812	117.108	61.378	7.829	117.001	61.48	7.778	116.9
I21				61.035	8.954	125.243			
N22	53.321	9.003	124.268	53.187	9.019	123.975	53.07	9.007	124.3
C23	56.179	9.485	122.855	56.132	9.502	123.273	55.92	9.450	122.5
K24	54.055	9.190	131.116	53.865	9.164	130.406			
S25	56.299	9.368	120.533	56.074	9.381	120.363	56.04	9.300	120.3
S26	61.008	8.440	117.031	60.746	8.450	116.918	60.73	8.417	117.0
Q27	53.999	7.328	117.580	53.840	7.369	117.718	53.66	7.284	117.4
S27a	58.428	8.721	117.926	58.306	8.736	118.021	58.16	8.703	117.9
V27b	59.623	8.171	118.554	59.381	8.228	118.829	59.34	8.167	118.6
L27c	54.802	7.606	125.364	54.457	7.473	125.120	54.47	7.494	125.4
Y27d	55.814	9.199	133.251	55.472	9.203	132.739			
S27e				61.562	8.911	124.790			
S27f	61.143			60.911					
N28	51.949	6.925	114.521	51.746	6.950	114.695			
S29	59.921	8.151	112.654	59.717	8.131	112.581	59.66	8.126	112.5
K30	55.011			54.531	7.938	118.770	54.34	7.879	118.5
N31	53.248	9.038	121.245	52.880	9.017	121.358	53.05	9.041	121.1
Y32	57.287	8.997	131.843	56.837					
L33				51.495	7.112	126.075	51.35	7.241	125.4
A34	49.709			49.566	9.456	130.389			
W35									
Y36	57.071	10.051	121.418	56.730	10.063	120.916			
Q37	54.038	9.440	123.730	53.708	9.379	123.471			
Q38	55.631	9.872	130.341	55.838	9.555	128.473			
K39		8.832	133.617	54.846	9.036	133.814			
P40	64.669								
G41	46.013	8.594	114.111	45.915	8.659	114.293			
Q42	53.510	8.006	121.144	53.230	8.023	121.166			

P43									
P44									
K45									
L46									
L47	55.085			54.924	9.059	125.694			
I48	58.164	7.329	118.626	57.609	7.311	118.431			
Y49				55.706					
W50				56.936					
A51				57.826	9.508	118.684	57.74	9.489	118.6
S52	58.306			57.915	8.787	113.500	57.95	8.718	113.4
T53	63.850	9.219	124.804	63.527	9.055	124.636	63.60	9.169	124.6
R54	57.112	8.893	128.476	56.791	8.792	129.404			
E55	55.714	8.090	125.493	55.327	8.187	125.889			
S56	60.452			59.957	9.560	121.737		8.042	126.2
G57	45.606	8.881	115.594	45.483	8.881	115.034	45.39	8.877	115.3
V58	60.863	7.601	125.771	60.421	7.591	125.522	60.52	7.582	125.7
P59	63.707			63.486			63.39		
D60	56.075	8.532	120.034	55.867	8.539	120.208	55.81	8.519	120.1
R61	57.053	7.072	114.897	56.818	7.074	114.838	56.76	7.036	114.8
F62	58.338	7.750	121.310	58.117	7.744	121.177	58.03	7.774	121.2
S63	57.280	8.945	116.333	57.273	8.939	116.210	57.12	8.888	116.0
G64	44.181	9.219	114.077	43.998	9.155	113.737	43.86	9.180	114.2
S65	57.308	9.243	117.204	57.066	9.212	117.161	57.04	9.221	117.2
G66	45.034	8.523	112.078	44.805	8.501	111.990	44.77	8.502	111.9
S67	57.859	7.092	109.438	57.602	7.107	109.615	57.60	7.069	109.3
G68	48.868	9.058	114.938	48.583	9.051	114.848	48.58	9.050	114.8
T69	60.759	7.825	115.504	60.550	7.857	115.551	60.48	7.819	115.5
D70	54.358	6.591	122.279	54.206	6.620	122.300	54.11	6.564	122.2
F71	57.732	8.968	122.459	57.555	8.940	122.687	57.47	8.950	122.4
T72	61.830	8.812	117.772	61.426	8.860	117.665	61.54	8.788	117.7
L73	53.437	8.859	130.283	53.246	8.809	130.164	53.16	8.815	130.3
T74	61.252	8.938	123.697	61.046	8.948	123.692	60.96	8.892	123.5
I75	60.156	8.866	128.230	59.959	8.877	128.265	60.06	8.833	128.1
S76	60.606	8.833	122.393	60.542	8.890	122.470	60.24	8.850	122.2
S77	56.672	6.663	114.366	56.445	6.592	114.337	56.41	6.631	114.2
L78	57.852	8.519	126.481	57.651	8.578	127.028	57.55	8.506	126.3
Q79	53.515	9.036	127.125	53.258	9.053	127.270	53.23	9.008	126.9
A80				55.929	9.016	125.858	55.88	8.963	125.6
E81	57.790	8.942	114.778	57.472	8.906	114.774	57.52	8.914	114.8
D82	54.703	8.105	121.000	54.487	8.119	120.871	54.40	8.075	120.8
V83	65.733	7.127	123.470	65.476	7.123	123.556	65.52	7.093	123.3
A84	51.774	8.038	130.118	51.558	8.015	130.035			
V85	61.766	7.741	118.920	61.363	7.819	119.092	61.51	7.756	118.8
Y86	57.210	8.776	125.544	56.563	8.622	124.388			
Y87	57.404	9.622	121.489	57.146	8.784	121.157			
C88	53.780	7.945	118.214	53.133	7.880	117.777			
Q89				53.992	8.979	123.653			
Q90				52.516	8.730	125.155			
Y91				53.869					
Y92				59.762	8.603	123.401			
S93				55.641	8.532	113.163			
T94									
P95									
Y96									
S97									

F98	57.573						56.81		
G99	45.203	8.769	107.587	44.790	9.048	108.962	44.75	8.940	108.3
Q100	57.799	9.048	118.197	57.630	8.616	114.446			
G101	45.659	6.923	108.393	45.405	7.079	108.245	45.19	6.901	108.1
T102	61.947	8.053	119.605	61.788	8.140	119.628			
K103	56.897	8.323	130.430	56.690	8.347	130.043	56.61	8.272	130.1
L104	54.620	8.748	131.212	54.343	8.705	131.008	54.42	8.702	131.4
E105	54.290	8.752	124.670	54.108	8.793	124.491	54.06	8.718	124.6
I106	59.940	8.340	120.574	59.745	8.347	120.504	59.63	8.307	120.5
K107	56.364	7.957	131.029	56.148	7.958	130.944	56.05	7.918	130.9
R108	57.793	8.215	133.647		8.216	133.649	57.53	8.191	133.5

Table S3. Association constants of LEN, SMA and SMA-like mutants of LEN. The association constants were obtained from non-linear fits of molar ellipticity at 282.5 nm as a function of protein concentration as described in the Methods section (c.f., Supplementary Fig. S2). The non-linear fitting routine did not converge for LEN Q89H.

Protein	Association Constant ($x\ 10^5\ M^{-1}$)
LEN	2.0 ± 0.2
SMA	12.8 ± 3.9
S29N	113 ± 10
K30R	6.5 ± 0.4
P40L	18.5 ± 2.8
T94H	2.7 ± 2.3
Y96Q	3.4 ± 0.3
S97T	1.0 ± 0.2
I106L	1.8 ± 0.1

Table S4. Summary of ^{15}N relaxation dispersion NMR data analysis for the three dynamic regions of LEN, SMA and SMA-like mutants of LEN.

	LEN[¶]	S29N	K30R	P40L	Q89H	T94H	Y96Q	S97T	I106l	SMA
					**Region 1**					
k_{ex} p_B	*200±40 s^{-1}, 4.0±0.4 %*	*60±15 s^{-1}, 9.0±1.9 %*	*60±22 s^{-1}, 8.0±2.3 %*	*340±53 s^{-1}, 3.0±0.5 %*	*289±18 s^{-1}, 51±13 %[c]*	*n/a*	*420±15 s^{-1}, 17.0±0.4 %*	*35±19 s^{-1}, 9.0±4.6 %*	*64±17 s^{-1}, 10.0±2.1 %*	*1100±80 s^{-1}, n/a*
	Δω (ppm)	Δω (ppm)	Δω (ppm)	Δω (ppm)	Δω (ppm)	Δω (ppm)	Δω (ppm)	Δω (ppm)	Δω (ppm)	Δω (ppm)
I2	–[a]	0.37±0.05[c]	-0.37±0.06	-0.66±0.03[c]	-0.26±0.01	–	n/a	1.0±0.08[c]	0.39±0.04	n/a
V3	-0.99±0.04	-1.00±0.04	-0.88±0.05	-0.93±0.03	-0.41±0.01	–	n/a	-0.57±0.07	-0.77±0.04	n/a
M4	-0.80±0.03	-0.78±0.04	-0.66±0.05	-0.79±0.03	-0.40±0.01	–	1.09±0.01	-0.41±0.07	-0.71±0.04	n/a
T5	-0.56±0.04[c]	-0.59±0.04	-0.47±0.05	-0.53±0.04[c]	-0.24±0.01[c]	–	-0.40±0.01	-0.56±0.06	-0.46±0.04	n/a
Q6	-1.26± 0.04	-1.43±0.06	-1.18±0.06	-1.12±0.04	-0.56±0.01	–	-2.54±0.02	-1.32±0.09	-1.27±0.04	n/a
S7	1.03±0.04	1.09±0.05[c]	-0.94±0.05[c]	0.96±0.03	-0.36±0.01	n/a	n/a	-1.1±0.09	1.04±0.04	n/a
P8	n/a[b]	n/a	n/a	n/a	n/a	n/a	n/a	n/a	n/a	n/a
D9	-0.18±0.09[c]	~0	~0	-0.29±0.06[c]	~0	–	0.39±0.01	~0	~0	n/a
					**Region 2**					
k_{ex} p_B	*630±30 s^{-1}, 2.3±0.06 %*	*500±21 s^{-1}, 3.2±0.1 %*	*290±24 s^{-1}, 2.6±0.2 %*	*620±28 s^{-1}, 2.2±0.1 %*	*92±9 s^{-1}, 10±1 %*	*n/a*	*260±17 s^{-1}, 6.8±0.3 %*	*550±38 s^{-1}, 1.6±0.1 %*	*410±22 s^{-1}, 2.7±0.1 %*	*850±70 s^{-1}, 1.8±0.8 %*
	Δω (ppm)	Δω (ppm)	Δω (ppm)	Δω (ppm)	Δω (ppm)	Δω (ppm)	Δω (ppm)	Δω (ppm)	Δω (ppm)	Δω (ppm)
Y32	–	-0.88±0.03	-1.12±0.05	-0.88±0.04	-4.36±0.11	n/a	n/a	-0.31±0.02	-0.98±0.04	n/a
L33	–	-0.86±0.03	-0.55±0.05	-0.84±0.04[c]	n/a	–	1.55±0.03[c]	-0.3±0.02	-0.75±0.04	n/a
A34	–	-0.30±0.06	-0.14±0.15	-0.19±0.12[c]	n/a	–	n/a	~0	-0.17±0.11	n/a
W35	–	-0.53±0.04	-0.48±0.05	-0.67±0.04	n/a	n/a	n/a	-0.18±0.03	-0.60±0.04	n/a
Y36	–	0.40±0.06	-0.16±0.12	-0.65±0.04[c]	-2.12±0.07	–	n/a	~0	~0	n/a
Q37	–	n/a	n/a	n/a	-1.37±0.04	–	n/a	n/a	n/a	n/a
Q38	–	-0.75±0.03	-0.64±0.05	-0.77±0.04[c]	-1.28±0.05	–	n/a	-0.15±0.05	-0.69±0.04	n/a
K39	–	-0.43±0.05[c]	-0.42±0.06	-0.67±0.04	-0.77±0.03	–	n/a	-0.10±0.06	-0.41±0.05	1.15±0.05[c]
P40[d]	n/a	n/a	n/a	n/a	n/a	n/a	n/a	n/a	n/a	0.69±0.06[c]
G41	–	-0.67±0.05	-1.0±0.05	-0.77±0.04	-0.79±0.05	–	n/a	-0.44±0.01	0.94±0.06	-1.03±0.04
Q42	1.88±0.04	1.71±0.03[c]	-1.69±0.06	2.05±0.05	-1.94±0.06	–	n/a	-0.52±0.02	-1.91±0.05[c]	-1.25±0.05
P43	n/a	n/a	n/a	n/a	n/a	n/a	n/a	n/a	n/a	n/a
P44	n/a	n/a	n/a	n/a	n/a	n/a	n/a	n/a	n/a	n/a
K45	1.99±0.06	1.8±0.04[c]	-1.86±0.08	1.88±0.05[c]	n/a	n/a	n/a	-0.53±0.02	1.72±0.05[c]	n/a
L46	2.57±0.06	2.42±0.06[c]	-2.75±0.12	2.31±0.06	n/a	n/a	n/a	-0.63±0.01	2.96±0.09	n/a
L47	n/a	n/a	0.58±0.05[c]	0.51±0.06	n/a	–	n/a	0.18±0.04[c]	-0.58±0.04	n/a
I48	-0.32±0.07	-0.32±0.05	-0.25±0.09	-0.4±0.06	-0.47±0.03[c]	–	n/a	~0	-0.26±0.07	0.93±0.11[c]

	LEN	S29N	K30R	P40L	Q89H	T94H	Y96Q	S97T	I106l	SMA
**Region 2, continued**										
Y49	-2.01±0.05	-1.68±0.04	-2.93±0.12	-2.02±0.05	n/a	n/a	n/a	-0.49±0.02	-2.02±0.06	n/a
W50	2.82±0.07	3.25±0.08	-2.06±0.08	3.43±0.10	n/a	n/a	n/a	0.76±0.01	3.24±0.14	n/a
A51	1.19±0.04	1.00±0.03	1.11±0.05	1.10±0.04	n/a	–	0.57±0.03[c]	0.34±0.02	1.12±0.04	n/a
S52	~0	~0	~0	~0	n/a	–	-0.46±0.02	~0	~0	~0
T53	-0.48±0.06	-0.44±0.04	-0.34±0.07	-0.61±0.05	-0.63±0.03	–	0.92±0.02[c]	~0	-0.39±0.05	1.84±0.06[c]
R54	1.78±0.04[c]	-1.58±0.03[c]	-2.16±0.08	1.85±0.05	2.85±0.10[c]	–	n/a	0.52±0.01[c]	1.85±0.05	n/a
E55	1.21±0.04	-1.10±0.03[c]	-1.1±0.05[c]	-1.02±0.04[c]	2.84±0.10[c]	–	2.81±0.06[c]	-0.36±0.02[c]	-1.11±0.04	n/a
S56	-2.18±0.06	-2.03±0.05	-2.46±0.10	-2.20±0.06	n/a	–	n/a	-0.54±0.01	-2.48±0.07	4.21±0.16[c]
G57	-1.39±0.04	-0.95±0.03	-1.02±0.04	-1.04±0.04	-0.86±0.03	–	1.17±0.02[c]	-0.34±0.02	-0.95±0.03	0.82±0.08[c]
V58	–	-0.40±0.04	-0.31±0.07	-0.40±0.07	-0.38±0.03	–	0.54±0.02	~0	-0.40±0.05	-0.62±0.06
P59	n/a	n/a	n/a	n/a	n/a	n/a	n/a	n/a	n/a	n/a
D60	n/a	-0.56±0.03	-0.43±0.05	-0.62±0.04[c]	-0.61±0.03	–	-0.88±0.02	-0.24±0.03	-0.52±0.04	0.78±0.05[c]
**Region 3**										
k_{ex} p_B	***250±30 s^{-1}, 2.3±0.21 %***	***170±24 s^{-1}, 3.5±0.4 %***	***110±27 s^{-1}, 3.9±0.8 %***	***530±54 s^{-1}, 4.4±1.4 %***	***540±33 s^{-1}, 4.7±0.2 %***	***n/a***	***n/a***	***330±60 s^{-1}, 1.8±0.2 %***	***310±35 s^{-1}, 3.5±0.2 %***	***1390±90 s^{-1}, n/a***
	Δω (ppm)	Δω (ppm)	Δω (ppm)	Δω (ppm)	Δω (ppm)	Δω (ppm)	Δω (ppm)	Δω (ppm)	Δω (ppm)	Δω (ppm)
C88	±	-0.42±0.05	-0.36±0.08	-0.14±0.08	-0.86±0.03	–	n/a	~0	-0.31±0.05	n/a
Q89[d]	-1.13±0.06[c]	-0.90±0.05	-0.75±0.06	0.71±0.02	n/a	–	n/a	~0	-0.72±0.03[c]	n/a
Q90	1.29±0.07[c]	1.20±0.05[c]	0.95±0.06[c]	0.73±0.02	n/a	–	n/a	-0.34±0.02[c]	-1.05±0.03	n/a
Y91	-0.70±0.06[c]	0.76±0.05[c]	-0.51±0.07	-0.56±0.03	n/a	n/a	n/a	-0.22±0.03	-0.68±0.04	n/a
Y92	-0.84±0.06	-0.78±0.05	-0.53±0.07	-0.52±0.03	n/a	–	n/a	~0	-0.54±0.04	n/a
S93	1.10±0.06[c]	1.08±0.05	-0.85±0.06	0.61±0.03	n/a	–	n/a	-0.25±0.03	-0.75±0.03[c]	n/a
T94[d]	-0.74±0.06	0.53±0.05[c]	-0.51±0.06	-0.60±0.03	n/a	n/a	n/a	-0.29±0.02	-0.43±0.04	n/a
P95	n/a	n/a	n/a	n/a	n/a	n/a	n/a	n/a	n/a	n/a
Y96[d]	n/a	n/a	n/a	n/a	n/a	n/a	n/a	n/a	n/a	n/a
S97[d]	2.88±0.12	2.09±0.07[c]	1.82±0.07[c]	n/a	n/a	n/a	n/a	n/a	n/a	n/a
F98	-2.02±0.09	0.94±0.05[c]	1.66±0.09[c]	n/a	n/a	n/a	n/a	0.47±0.01[c]	-1.26±0.04	n/a
G99	1.67±0.07	1.60±0.06	1.74±0.09	-1.0±0.02[c]	-1.51±0.05	–	–	-0.50±0.01	1.47±0.04	n/a
Q100	-2.92±0.15	-2.4±0.1	-2.37±0.13	-0.71±0.02[c]	-2.11±0.08	–	n/a	-0.35±0.02	-1.01±0.03	n/a
G101	-0.44±0.07[c]	-0.46±0.05[c]	-0.52±0.06	-0.27±0.04	-0.67±0.03	–	–	-0.16±0.04	-0.36±0.05	n/a
T102	–	0.53±0.05[c]	0.33±0.08	0.39±0.03	0.72±0.03	–	n/a	~0	n/a	n/a

[*]The signs of chemical shift differences were obtained according to the method of Kay and co-workers [7].
[¶]Values taken from previous work [2].

[a]Resonance present in spectrum but not included in data analysis due to low R_{ex} value.
[b]Resonance not included in analysis due to excessive exchange broadening, spectral overlap, a proline residue, inability to reliably extract Δω or the simulation not converging.
[c]Difference in ^{15}N resonance frequencies between HSQC and HMQC spectra < 0.3 Hz.
[d]Residue identity differs for SMA and SMA-like LEN mutants.
[e]Note that although the unrestricted simulation of residues in region 1 for LEN Q89H consistently yielded a high value for p_B and relatively small Δω values, such a high population of minor conformer is unlikely. Indeed, by fixing the residue specific Δω values to the average of those obtained for the other LEN mutants, a comparable quality fit of the experimental data for LEN Q89H region 1 is obtained with the following parameters: p_B = 11.2 ± 1 % and k_{ex} = 112 ± 12 s^{-1}, in line with those obtained for the other mutants.

References

1 Farrow, N. A. *et al.* Backbone dynamics of a free and a phosphopeptide-complexed Src homology 2 domain studied by ^{15}N NMR relaxation. *Biochemistry* **33**, 5984-6003 (1994).

2 Mukherjee, S., Pondaven, S. P. & Jaroniec, C. P. Conformational flexibility of a human immunoglobulin light chain variable domain by relaxation dispersion nuclear magnetic resonance spectroscopy: Implications for protein misfolding and amyloid assembly. *Biochemistry* **50**, 5845-5857 (2011).

3 Huang, D. B. *et al.* Variable domain structure of κIV human light chain Len: High homology to the murine light chain McPC603. *Mol. Immunol.* **34**, 1291-1301 (1997).

4 Wilkins Stevens, P. *et al.* Recombinant immunoglobulin variable domains generated from synthetic genes provide a system for in vitro characterization of light-chain amyloid proteins. *Protein Sci.* **4**, 421-432 (1995).

5 Kabat, E. A., Wu, T. T., Perry, H. M., Gottesman, K. S. & Foeller, C. Sequences of proteins of immunological interest, 5th ed. , NIH publication No. 91-3242, U.S. Department of Health and Human Services, Washington, DC (1991).

6 Baden, E. M. *et al.* Altered dimer interface decreases stability in an amyloidogenic protein. *J. Biol. Chem.* **283**, 15853-15860 (2008).

7 Skrynnikov, N. R., Dahlquist, F. W. & Kay, L. E. Reconstructing NMR spectra of "invisible" excited protein states using HSQC and HMQC experiments. *J. Am. Chem. Soc.* **124**, 12352-12360 (2002).
